# Circadian and behavioral timing in MASLD: a systematic review of human observational studies

**DOI:** 10.1016/j.eclinm.2026.104187

**Published:** 2026-08-29

**Authors:** Arunkumar Subramanian, Raja Affendi Raja Ali, Gavin Stewart Dawe, Vetriselvan Subramaniyan

**Affiliations:** a Faculty of Medical and Life Sciences, Sunway University, Selangor, Malaysia; b Department of Pharmacology, Yong Loo Lin School of Medicine, National University of Singapore, Singapore; c Faculty of Health Sciences, Villa College, Malé, Maldives

**Keywords:** Circadian disruption, MASLD, Shift work, Chronotype, Meal timing

## Abstract

**Background:**

Circadian disruption may affect metabolism through altered timing of sleep, activity, light exposure, hormonal rhythms, and food intake. However, its relationship with metabolic dysfunction-associated steatotic liver disease (MASLD), including fibrosis and cirrhosis remains unclear. To evaluate associations between circadian and behavioural timing exposures and MASLD in observational studies.

**Methods:**

PubMed, Embase, and Scopus were searched from inception to 14 July 2026. Eligible studies examined naturally occurring timing-related exposures in adults and reported steatosis, fibrosis, cirrhosis, or quantitative liver-fat outcomes. Findings were synthesized narratively because heterogeneity precluded pooling. Risk of bias was assessed using AXIS or the Newcastle–Ottawa Scale, and certainty using an adapted domain-level GRADE approach (PROSPERO Registration number: CRD420261381636).

**Findings:**

Twenty-four independent MASLD-related evidence sources were included in the primary synthesis; two additional mixed-aetiology cirrhosis studies were considered contextually. Shift work showed the most consistent associations and most longitudinal evidence. Chronotype was generally associated with disease occurrence but inconsistently with advanced outcomes: intermediate or late chronotype was associated with fibrosis in one clinical cohort, whereas morning chronotype was not independently associated with incident cirrhosis in a prospective cohort. Later sleep timing was associated with prevalent MASLD and, in one NHANES analysis, fibrosis. Evidence for objective rest-activity rhythms, light exposure, meal timing, and melatonin phase remained limited or heterogeneous. Mixed-aetiology cirrhosis studies suggested that advanced liver disease may itself disrupt circadian physiology.

**Interpretation:**

Circadian and behavioural timing exposures showed trends associated with MASLD-related outcomes, with the most consistent evidence for shift work. Residual confounding, cross-sectional designs, self-reported exposures, heterogeneous outcomes, and reverse causality preclude causal conclusions. Prospective studies using objective circadian phenotyping and standardized fibrosis outcomes are needed.

**Funding:**

This research did not receive any specific grant from funding agencies in the public, commercial, or not-for-profit sectors.

**Research in context**

**Evidence before this study**

We searched PubMed, Embase, and Scopus from inception to July 14, 2026, for human observational studies examining circadian or behavioural timing exposures in relation to metabolic dysfunction-associated steatotic liver disease (MASLD), including steatosis, fibrosis, cirrhosis, and quantitative liver-fat outcomes. Search terms covered shift work, chronotype, social jetlag, irregular or delayed sleep, rest–activity rhythms, nocturnal light exposure, endogenous melatonin, meal timing, and related MASLD and liver-disease outcomes; the complete database-specific search strategies and exact search terms are provided in Supplementary Tables S2–S4 for PubMed, Scopus, and Embase, respectively. Previous evidence was dispersed across occupational health, sleep epidemiology, chrononutrition, and circadian-biology research. These constructs were frequently assessed separately and were sometimes conflated with general sleep duration or sleep quality. Most studies were cross-sectional, exposure and liver-outcome definitions varied substantially, and adjustment for adiposity, metabolic factors, occupational characteristics, and socioeconomic conditions was inconsistent. Evidence concerning fibrosis, cirrhosis, objective circadian measures, and the direction of causality was particularly limited.

**Added value of this study**

This systematic review integrates 24 independent MASLD-related evidence sources within a unified framework of five distinct exposure domains: shift work; chronotype; sleep timing, sleep regularity, and circadian misalignment; objective rest-activity, light, and circadian biomarker measures; and meal timing. Two additional mixed-aetiology cirrhosis studies were considered separately to examine whether advanced liver disease may itself disturb circadian physiology. The review distinguishes chronotype as an individual timing phenotype from shift work as an externally imposed, multidimensional occupational exposure, and separates realized sleep timing and regularity from endogenous circadian phase. It also incorporates advanced fibrosis and cirrhosis outcomes, links overlapping reports to avoid double counting, evaluates risk of bias using design-appropriate tools, and assesses certainty by exposure domain. Statistical pooling was not undertaken because exposure definitions, comparators, outcomes, and analytical models were too heterogeneous for a clinically meaningful summary estimate. Shift work had the largest and most consistent evidence base and the greatest longitudinal support, whereas evidence for the other domains remained limited, inconsistent, or predominantly cross-sectional.

**Implications of all the available evidence**

Circadian and behavioural timing may represent an important temporal dimension of MASLD risk, but the current evidence does not establish an independent causal relationship. Shift work should be interpreted as a complex exposure involving changes in sleep, light, meal timing, activity, occupational stress, and social routines rather than as a direct measure of endogenous circadian disruption. Current evidence is insufficient to support validated circadian screening tools or circadian-targeted treatment recommendations for MASLD. Future studies should use prospective designs, repeated and objective circadian phenotyping, standardized imaging or histological liver outcomes, robust confounder control, sex-stratified analyses, and explicit differentiation between MASLD and MetALD. These priorities are necessary to determine whether timing-related exposures improve risk prediction or represent modifiable therapeutic targets.

## Introduction

Metabolic dysfunction-associated steatotic liver disease (MASLD), formerly classified under non-alcoholic fatty liver disease (NAFLD), has become one of the most important metabolic liver conditions worldwide. The updated MASLD nomenclature emphasizes the presence of hepatic steatosis in the context of cardiometabolic risk, thereby placing liver disease within a broader systemic metabolic framework rather than treating it as an isolated hepatic disorder.^1^^,^^2^ MASLD is closely linked with obesity, insulin resistance, dysglycaemia, dyslipidemia, and cardiometabolic morbidity, and its global prevalence has continued to rise in parallel with modern lifestyle-related metabolic disease.^3^ Although current prevention and management strategies appropriately emphasize weight loss, dietary quality, physical activity, and metabolic risk-factor control, these approaches largely focus on what and how much individuals eat or move, while giving comparatively less attention to when these behaviours occur and whether they are aligned with endogenous biological rhythms.

Circadian rhythms coordinate approximately 24-h cycles in sleep-wake behaviour, feeding-fasting patterns, hormone secretion, glucose handling, lipid metabolism, inflammation, and energy homoeostasis.^4^^,^^5^ Importantly, the liver is not merely a passive target of systemic metabolism; it contains a robust peripheral clock that regulates temporally organized hepatic functions, including nutrient processing, lipid handling, bile acid metabolism, detoxification, and inflammatory signalling.^6^ Disruption may occur when behavioural or environmental timing conflicts with endogenous rhythms. Shift work can impose wakefulness, light exposure, activity, sleep, and food intake during biologically inappropriate phases, whereas chronotype reflects a relatively stable preference for earlier or later sleep and activity timing. Evening chronotype is not inherently pathological; risk may arise when preferred timing conflicts with occupational or social schedules. Irregular sleep timing, social jetlag, nocturnal light exposure, and mis-timed food intake provide additional pathways to misalignment.

These disturbances are relevant to MASLD because pathways involved in hepatic steatosis and disease progression are circadian-regulated. Experimental and human laboratory studies show that circadian misalignment can impair glucose regulation, insulin sensitivity, and alter blood pressure, cortisol, leptin, and inflammatory markers.^7^^,^^8^ Circadian disruption may therefore interact with established metabolic pathways-including hepatic lipid accumulation, insulin resistance, inflammation, mitochondrial stress, and altered substrate flux, rather than act as an isolated cause.

Clinical evidence nevertheless remains fragmented across occupational, sleep, chrononutrition, and objective circadian research. Studies have examined shift work, chronotype, social jetlag, sleep irregularity, meal timing, actigraphy, light exposure, and circadian biomarkers. These related but distinct constructs differ in exposure definition, comparator structure, study design, liver-outcome ascertainment, and covariate adjustment.

A systematic synthesis is therefore needed to distinguish general sleep traits from specifically circadian or timing-related exposures and to organize the heterogenous evidence into biologically meaningful domains. The available evidence spans diverse exposure domains, outcomes, and study designs, making statistical pooling potentially misleading. Accordingly, this systematic review evaluated associations between circadian and behavioural timing exposures and the occurrence or progression of MASLD in human observational studies. Five exposure domains were examined: shift work, chronotype, sleep timing, sleep regularity, and circadian-misalignment constructs, objective rest–activity, light and circadian biomarker measures, and meal timing. The direction, consistency, methodological quality, and certainty of evidence were assessed to determine whether biological or behavioural timing represents an important temporal dimension of MASLD risk.

## Methods

### Protocol and registration

This systematic review was conducted in accordance with the Preferred Reporting Items for Systematic Reviews and Meta-Analyses (PRISMA) 2020^9^ statement, and was registered in PROSPERO (CRD420261381636).^10^ The review question, eligibility framework, exposure domains, and synthesis methods were defined before study selection. The review focused on human observational evidence and used narrative synthesis because the eligible studies differed substantially in exposure definition, comparator structure, liver-outcome ascertainment, and reported effect measures.

### Review question and eligibility framework

This review evaluated associations between circadian and behavioural timing exposures and the occurrence or progression of MASLD or equivalent fatty liver outcomes in adults using a PECO framework. The population comprised adults from community, occupational, or clinical settings. Exposures included shift or night work; chronotype; sleep timing and regularity; social jetlag or other study-defined circadian-misalignment constructs; objective rest–activity or light-related rhythms; endogenous circadian biomarkers; and meal-timing disruption. Comparators were lower or absent exposure, alternative exposure categories, or reference timing patterns defined by the original studies. Eligible outcomes included prevalent or incident MASLD, MAFLD, or NAFLD; hepatic steatosis; significant or advanced fibrosis; cirrhosis; other severity outcomes; and quantitative liver-fat measures assessed by imaging, elastography, biopsy, validated administrative coding, or accepted disease-oriented indices.

Eligible studies included adult human participants, assessed a clinical, behavioural, occupational, environmental, or biomarker-based indicator of circadian disruption or behavioural timing, reported a MASLD-, MAFLD-, or NAFLD-related liver outcome, used an observational design, and provided sufficient information to determine the direction of association. Eligible designs included cross-sectional, case-control, prospective or retrospective cohort, longitudinal, and nested observational analyses. Studies without an external comparator were retained only as descriptive evidence and were not interpreted as comparative association studies.

Studies of advanced fibrosis or cirrhosis were included in the primary synthesis when the underlying liver disease was explicitly attributed to MASLD, MAFLD, or NAFLD, or when a relevant subgroup was separately extractable. Studies restricted to viral, alcohol-associated, autoimmune, cholestatic, mixed, unspecified, or other non-MASLD liver disease were excluded unless metabolic steatotic liver-disease data were reported separately. Selected mixed-aetiology cirrhosis studies were retained only to examine whether established advanced liver disease may itself disturb circadian physiology and were not counted as evidence that circadian disruption preceded MASLD progression.

Intervention studies assigning time-restricted eating, intermittent fasting, melatonin, light exposure, sleep scheduling, shift-schedule modification, or other circadian-targeted treatments were excluded because the review focused on naturally occurring exposures. Mendelian-randomization, bioinformatics, and gene-expression studies without direct clinical exposure-outcome analysis were also excluded. General sleep duration, insomnia, sleep quality, obstructive sleep apnea, composite constructs without an interpretable circadian exposure, and isolated liver-enzyme abnormalities were ineligible unless a distinct timing-related exposure and qualifying liver outcome were reported.

Studies using endogenous melatonin were excluded when specimen-collection time or conditions were unreported, or when the assay method could not be identified or verified. Isolated absolute concentrations without adequate temporal and analytical information were not considered interpretable measures of circadian phase, amplitude, or rhythmicity. Concentrations were not compared across studies when biological matrix, clock time, light conditions, assay platform, or sampling protocol differed. Conference abstracts, reviews, editorials, commentaries, letters without original data, animal studies, and in-vitro studies were also excluded. When publications included overlapping participants, they were linked as one underlying evidence source. The most comprehensive report was used to describe the population, while unique exposures or outcomes from companion reports were retained without being counted as independent replication. The complete eligibility criteria are presented in Supplementary Table S1.

### Information sources

A systematic literature search was conducted in PubMed, Embase, and Scopus from database inception to 14th July, 2026. Reference lists of included studies and relevant reviews were also screened to identify additional eligible reports.

### Search strategy

The search strategy combined controlled vocabulary terms, where applicable, and free-text terms related to MASLD, MAFLD, NAFLD, steatohepatitis, hepatic fibrosis, advanced fibrosis, and cirrhosis with terms for circadian and behavioural timing exposures. Exposure concepts included circadian disruption or misalignment, chronotype, morningness or eveningness, social jetlag, shift work, night work, rotating shifts, sleep timing or irregularity, rest-activity rhythm, actigraphy, endogenous melatonin, meal timing, late eating, night eating, and timing of food intake. Search strategies were adapted to the syntax of each database and are provided in Supplementary Tables S2–S4.

### Study selection

Records were deduplicated and screened in Rayyan^11^ in two stages: title/abstract screening followed by full-text review. One mutually exclusive primary exclusion reason was assigned to each excluded full text. Eligibility disagreements were resolved by consensus between V.S. and R.A.R.A., with A.S. consulted when required. The process is shown in the PRISMA flow diagram.

### Data extraction

A structured form captured study setting/design, population and sample size, exposure definition and ascertainment, comparator, liver outcome and measurement, adjusted covariates, effect estimates with 95% confidence intervals, and relevant subgroup or severity analyses. For circadian biomarkers, we additionally recorded specimen, sampling time/conditions, light and fasting conditions, assay details, units, and repeated or phase-marker measurements.

Additional interpretive information included attenuation after adiposity adjustment, indirect outcome definitions, study sex composition, availability of sex-disaggregated or sex-stratified estimates, and whether sex was addressed through adjustment, stratification, or interaction testing. We also recorded whether studies assessed gender identity, gender roles, or gender-related social factors. No included study directly measured these constructs; therefore, “sex” is used when referring to male and female participant categories.

Studies contributing to multiple exposure domains were assigned a primary domain for tabular and graphical presentation, while all relevant findings were retained in the narrative synthesis. Reports with overlapping populations were linked and counted as one evidence source. This applied to the Weng et al.^12^ and Zong et al.^13^ NHANES analyses, which used overlapping survey cycles but examined different circadian constructs and liver outcomes.

### Exposure domains

Eligible studies were grouped into five prespecified exposure domains: shift-work-related exposures; chronotype; sleep timing, sleep regularity, and circadian-misalignment constructs beyond sleep duration; objective rest–activity, light, and circadian biomarker measures; and meal-timing-related exposures.

Chronotype was defined as a relatively stable tendency toward earlier or later preferred timing of sleep and activity, assessed using validated questionnaires, corrected midsleep measures, or study-defined morningness-eveningness categories. Sleep timing referred to observed bedtime, wake time, or sleep midpoint, whereas sleep regularity referred to day-to-day variability in these measures. Social jetlag described differences between workday and free-day sleep timing. Circadian misalignment was reserved for mismatch between endogenous circadian timing and externally imposed behavioural or environmental schedules, or for explicitly labelled composite constructs used by original investigators.

These constructs were analysed separately because chronotype, actual sleep timing, sleep regularity, and circadian misalignment are related but not interchangeable. When studies used broad or non-standard terminology, the operational exposure components were reported rather than accepting the authors’ label as an established chronobiological construct. This framework guided the narrative synthesis and visual summaries. Because the domains represented biologically and methodologically distinct constructs, their associations were not compared as equivalent exposure intensities, and chronotype evidence was not directly ranked against shift-work evidence. Some studies assessed more than one exposure; therefore, domain counts were not mutually exclusive.

Shift work was considered separately because it represents an externally imposed occupational schedule rather than an intrinsic timing phenotype. Night or rotating work may simultaneously alter sleep opportunity, light exposure, meal timing, physical activity, occupational stress, and social behaviour. It was therefore interpreted as a multidimensional exposure capable of inducing circadian misalignment, but not as synonymous with it. Chronotype was interpreted as a potential susceptibility or effect-modifying characteristic rather than an environmental exposure of equivalent intensity.

### Outcomes

Outcomes were grouped into four categories: prevalent disease/occurrence, incident disease, severity/fibrosis-related outcomes, and specialized liver phenotypes. Prevalent disease/occurrence included prevalent MASLD/MAFLD/NAFLD or hepatic steatosis. Incident disease referred to new-onset MASLD/MAFLD/NAFLD identified during follow-up. Severity/fibrosis-related outcomes included advanced steatosis, fibrosis, cirrhosis, or severity within established disease. Specialized liver phenotypes included hepatic fat fraction and other related quantitative liver measures. Outcome ascertainment methods across the included studies included ultrasonography, transient elastography or vibration-controlled transient elastography, biopsy, MRI-based liver fat quantification, ICD-based case identification, and validated disease indices used by the original studies.

### Risk of bias assessment

Risk of bias was assessed with AXIS^14^ for cross-sectional studies/components and the Newcastle-Ottawa Scale^15^ (NOS) for cohort and case-control studies. Mixed-design studies were appraised by component, and overlapping reports were assessed separately when methods differed but linked at evidence-source level. AXIS items were not summed because no validated total score or cut-off exists; review-level judgements emphasized sampling/representativeness, exposure and outcome measurement, non-response, and reporting completeness/internal consistency. Covariate control was evaluated separately because AXIS lacks a dedicated confounding domain. Detailed AXIS judgements and decision rules are in Supplementary Tables S5 and S5A, and NOS assessments in Supplementary Tables S6 and S7.

### Statistical analysis

No meta-analysis or statistical pooling was performed because the included studies differed substantially in exposure definitions, comparator structures, study designs, liver-outcome definitions, ascertainment methods, covariate adjustment, and effect measures. Pooling these estimates would therefore have produced a summary effect without clear clinical meaning.

Findings were synthesized narratively across five prespecified exposure domains. Within each domain, studies were compared by design, population, exposure and comparator definitions, liver-outcome ascertainment, direction and magnitude of associations, precision, confounder adjustment, and methodological limitations. The synthesis assessed consistency within and across domains but did not calculate pooled effects, statistical heterogeneity, or summary association measures. Risk-of-bias judgements informed the interpretive weight assigned to each study. High-risk studies were retained for completeness but were not used as the sole basis for domain-level conclusions. Greater weight was given to prospective designs, clearer sampling and comparator structures, validated exposure and outcome measures, and more comprehensive adjustment. No study was excluded solely on the basis of risk of bias.

Subgroup analysis and meta-regression were not undertaken because too few studies were clinically comparable within specific exposure-outcome combinations. Differences in exposure operationalization, reference categories, outcome definitions, adjustment models, and effect measures further prevented clinically coherent pooled comparisons. Heterogeneity was therefore examined descriptively rather than statistically.^16^

The primary synthesis was restricted to MASLD-related populations and outcomes. Observational studies of naturally occurring circadian or behavioural timing exposures were distinguished from intervention studies assessing deliberate modification of sleep, light, work, or meal timing. Because these designs differ in exposure allocation, comparator structure, bias, and interpretation, intervention studies were not incorporated into the domain-level synthesis.

Studies of circadian abnormalities in established mixed-aetiology cirrhosis were analysed separately to inform directionality and reverse causality. They were excluded from primary domain counts, certainty ratings, and interpretations of circadian disruption as an antecedent MASLD risk factor. Interpretation emphasized direction, consistency, temporality, and methodological limitations. Longitudinal and cross-sectional findings were considered separately, with explicit attention to reverse causality, residual confounding, exposure misclassification, and overlapping behavioural pathways. These considerations were used to contextualize the evidence rather than assign causal status to any exposure domain.

### Data presentation

Two descriptive visualizations were used: a domain-level evidence matrix and a study-level harvest plot. Matrix symbols ranged from no evidence (—) to comparatively extensive/coherent evidence (+++) and reflected evidence volume, design, replication, outcome coverage, consistency, and major limitations. They did not represent effect magnitude, statistical significance, risk of bias, or GRADE certainty. The harvest plot summarized association direction, design, sample-size category, and selected methodological features.

Because sex representation varied across occupational and population-based studies, the distribution of male and female participants and the availability of sex-stratified estimates or interaction analyses were summarized in Supplementary Table S9. This table identified reporting and representation gaps but was not used to infer sex-specific effects when formal effect-modification analyses were unavailable. Gender-specific evidence was not presented because no included study directly assessed gender identity, gender roles, or gender-related social exposures.

### Certainty of evidence

Certainty was assessed at the exposure-domain level using an adapted GRADE approach.^17^ All observational domains started at low certainty and were considered for downgrading for risk of bias, inconsistency, indirectness, imprecision, and suspected publication bias, or upgrading for large associations, exposure-response patterns, or residual confounding likely to attenuate an effect. Ratings were high, moderate, low, or very low.

### Role of funding source

This research did not receive any specific grant from funding agencies in the public, commercial, or not-for-profit sectors.

## Results

### Study selection

**Fig. 1:**
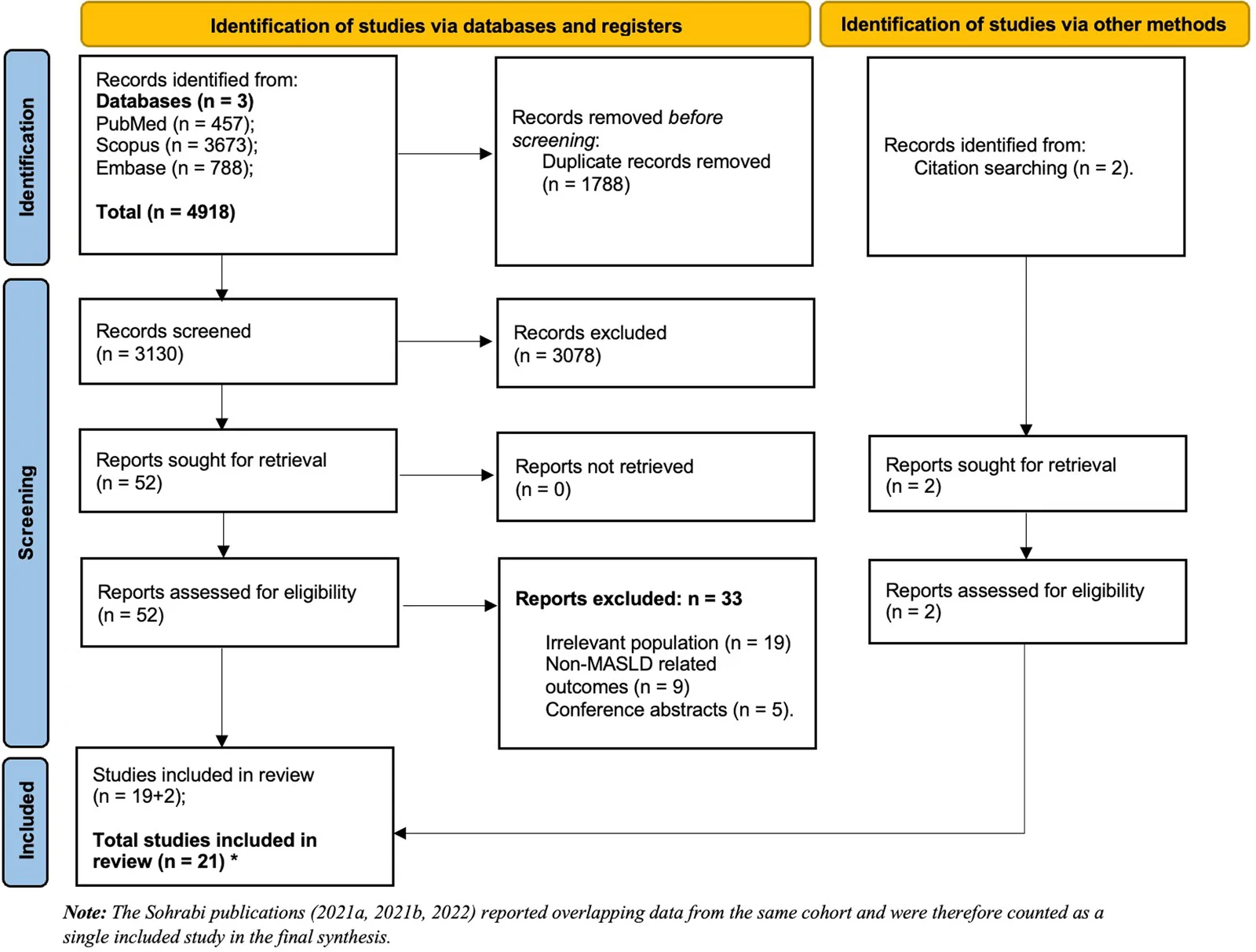
**PRISMA flow diagram.** Twenty-seven reports were retained after full-text assessment: 25 primary reports representing 24 independent MASLD-related evidence sources and two mixed-aetiology cirrhosis reports used only to contextualize reverse causality. “Ineligible population or liver-disease aetiology” referred to reports outside the adult-human eligibility framework or studies restricted to non-MASLD, mixed, or unspecified liver disease without separately extractable MASLD-related data. Reports involving eligible populations but lacking a MASLD-related liver outcome were classified separately as “non-MASLD-related outcome”. Weng et al. and Zong et al. used overlapping NHANES cycles and were treated as one underlying evidence source rather than as independent studies.

The search identified 10,491 records (PubMed 979; Scopus 7973; Embase 1539). After 2100 duplicates were removed, 8391 records were screened and 8312 excluded. Seventy-nine database reports and four from citation searching underwent full-text review. Fifty-six were excluded: 29 for ineligible population/liver-disease aetiology, 16 for no qualifying MASLD-related outcome, eight conference abstracts, and three overlapping reports with uninterpretable circadian biomarker measurement. Twenty-seven reports were retained: 25 reports (Table 1) representing 24 independent MASLD-related evidence sources for the primary synthesis and two mixed-aetiology cirrhosis studies for reverse-causality context (Fig. 1). Weng et al. and Zong et al. were treated as one overlapping NHANES evidence source.

**Table 1:** Characteristics of included studies.

Study	Country	Study design	Population/setting	Sample size	Primary exposure domain	Exposure definition	Comparator	Liver outcome	Outcome ascertainment	Main association/result
Chatur et al., 2024^18^	India	cross-sectional	security guards working rotating shifts	73	Shift work	rotating shift work	No external comparator	prevalent NAFLD	Ultrasonography	NAFLD prevalence 76.1%; no adjusted comparative estimate
Che et al., 2024^19^	China	cross-sectional analysis	female nurses in a tertiary hospital	824	Shift work	monthly night shift >5/month	≤5 night shifts per month	prevalent NAFLD	Ultrasonography	OR 3.512 (95% CI 1.280–9.633)
Chen et al., 2026^20^	United States	cross-sectional	US adults aged ≥20 years	4803	Sleep regularity/schedule discrepancy	weekend catch-up sleep >1 h	≤1 h weekend catch-up sleep	prevalent MASLD	VCTE/FibroScan	OR 1.38 (95% CI 1.14–1.67)
Fu et al., 2023^21^	China	longitudinal cohort	employed adults in a railway bureau	1309	Chronotype	evening chronotype	morning chronotype	incident MAFLD	abdominal ultrasonography	OR 2.19 (95% CI 1.09–4.40)
Gu et al., 2023^22^	United States	cross-sectional	American adults with overweight/obesity	4502	Objective rhythm/light/biomarker	relative amplitude	lowest quintile	prevalent suspected NAFLD	ALT-based proxy definition	OR 0.71 (95% CI 0.55–0.91)
Hu et al., 2026^23^	China	cross-sectional	middle-aged and older adults (≥40 years)	744	Chronotype	evening chronotype	morning chronotype	prevalent NAFLD	Ultrasonography	OR 1.70 (95% CI 1.03–2.81)
Huang et al., 2023^24^	United Kingdom	prospective cohort	UK Biobank participants in paid or self-employment	281,280	Shift work	usual/permanent night shift work	never/rarely worked night shifts	incident NAFLD	ICD-10 coding	HR 1.27 (95% CI 1.08–1.48)
Kani et al., 2023^25^	Türkiye	cross-sectional	biopsy-proven MASLD clinical cohort	93	Chronotype	non-morningness chronotype	morningness chronotype	advanced fibrosis	Biopsy	No significant association for chronotype; poor sleep quality OR 3.81 (95% CI 1.14–12.75)
Kim et al., 2022^26^	South Korea	cross-sectional	male steelworkers	2511	Shift work	shift work	daytime workers	moderate-to-severe NAFLD	Ultrasonography	OR 1.449 (95% CI 1.028–2.043)
Lee and Lee, 2024^27^	South Korea	longitudinal cohort	healthy Korean workers aged 20–59 years	45,149	Shift work	shift work	daytime workers	incident NAFLD	Ultrasonography	IRR 1.24 (95% CI 1.08–1.43) among participants in their 20s
Maidstone et al., 2024^28^	United Kingdom	cross-sectional	UK Biobank employed adults	282,303	Shift work	irregular shift work	day workers	prevalent NAFLD	DSI (indirect); MRI-PDFF subset	OR 1.12 (95% CI 1.03–1.22)
Peng et al., 2025^29^	China	cross-sectional	subway workers in Wuhan	9105	Shift work	shift work duration >6 years	no shift work	prevalent MAFLD	abdominal ultrasonography	OR 1.60 (95% CI 1.37–1.88)
Shi et al., 2025^30^	China	prospective cohort	adults in the Kailuan cohort	32,030	Meal timing	breakfast skipping + late-night snacking	regular breakfast and no late-night snacking	incident MASLD	abdominal ultrasonography	HR 1.52 (95% CI 1.30–1.77)
Vetrani et al., 2022^31^	Italy	cross-sectional	adults with obesity (BMI ≥35 kg/m^2^)	87	Chronotype	evening chronotype	morning chronotype	NAFLD severity	indirect indices (HSI, LFE, ION)	Higher ION score in evening chronotype (p = 0.014)
Schaeffer et al., 2024^32^	Switzerland	case-control study	biopsy-proven MASLD clinical cohort	51	Objective rhythm/light/biomarker	Actigraphy derived sleep-wake measures and serial salivary dim-light melatonin-onset assessment	healthy controls	biopsy-proven MASLD severity	liver biopsy; FibroScan	No clear DLMO or melatonin-profile difference; greater wakefulness after sleep onset (45.4 versus 21.3 min; p = 0.0004) and lower sleep efficiency in MASLD
Wu et al., 2025^33^	United Kingdom; United States	prospective cohort and cross-sectional analyses	general adults (UK Biobank and NHANES)	98,602	Objective rhythm/light/biomarker	relative amplitude	per 0.1-unit increase	incident (UK Biobank) and prevalent (NHANES) MASLD	ICD-10 coding; FLI/USFLI	HR 0.70 (95% CI 0.65–0.75) per 0.1-unit increase
Xu et al., 2023^34^	China	longitudinal cohort	Chinese rail workers	14,112	Shift work	frequent shift work (>3 times per week)	seldom shift work (<1 time per week)	incident NAFLD	abdominal ultrasonography	HR 1.179 (95% CI 1.059–1.312)
Zhang et al., 2025^35^	China	cross-sectional	male workers in the automobile manufacturing industry	4672	Shift work	cumulative night-shift hours >2920	≤730 h of night shifts	prevalent NAFLD	abdominal ultrasonography	Shift work OR 1.35 (95% CI 1.09–1.68); cumulative night-shift hours >2920 versus ≤730 h OR 1.64 (95% CI 1.23–2.17)
Zhang et al., 2020^36^	China	cross-sectional	male steelworkers in North China	6881	Shift work	current rotating night shift work status	never worked night shifts	prevalent NAFLD	abdominal ultrasonography	Current rotating night shift work OR 1.23 (95% CI 1.02–1.48); >7 night shifts/month OR 1.24 (95% CI 1.06–1.45); >8 h/night OR 1.27 (95% CI 1.08–1.51)
Castelnuovo et al., 2023^37^	Italy	Cross-sectional	Adults with NAFLD attending a tertiary metabolic liver-disease clinic	126	Chronotype	Intermediate or late chronotype based on MCTQ-derived corrected midsleep on free days	Early chronotype	Significant fibrosis (F ≥ 2) and advanced fibrosis (F ≥ 3)	FibroScan; ≥7.1 kPa for F ≥ 2 and ≥8.8 kPa for F ≥ 3	Intermediate/late chronotype: adjusted OR 9.29 (95% CI 1.33–64.50) for F ≥ 2 and OR 23.38 (95% CI 1.63–335.10) for F ≥ 3; estimates were highly imprecise
Qin et al., 2024^38^	United Kingdom	Prospective cohort	UK Biobank participants with baseline NAFLD	112,196	Chronotype	Morning chronotype; composite healthy-sleep score analysed separately	Non-morning chronotype	Incident cirrhosis	Linked hospital and mortality ICD records	Morning chronotype was not independently associated with cirrhosis: HR 0.92 (95% CI 0.78–1.08). A favourable composite sleep score was associated with lower risk, HR 0.55 (95% CI 0.39–0.78), but incorporated several non-circadian sleep traits
Tosoratto et al., 2025^39^	Spain	Cross-sectional occupational study	Employed adults undergoing occupational health assessment	53,053	Shift work	Shift work versus non-shift work	Non-shift workers	Elevated surrogate MASLD and fibrosis-risk indices	FLI, HSI, ZJU, FLD, FSI, LAP, and BARD	Shift work was associated with higher probabilities of elevated indices, including HSI OR 7.83 (95% CI 7.40–8.26) and ZJU OR 5.91 (95% CI 5.60–6.22)
Weng et al., 2021^12^ & Zong et al., 2025^13^	United States	Cross-sectional analyses	NHANES 2017–2018 in Weng; NHANES 2017–March 2020 in Zong	1786 in Weng; 5368 in Zong	Sleep timing, sleep regularity, and study-defined circadian misalignment	Weng: study-defined composite of mistimed, late, and irregular sleep. Zong: workday/free-day sleep timing, social jetlag, and sleep debt. These measures reflect behavioural sleep timing and regularity, not chronotype.	No circadian misalignment or reference timing categories	Prevalent MAFLD/MASLD and liver fibrosis	VCTE/FibroScan using CAP and liver-stiffness measurements	Weng: circadian misalignment OR 1.893 (95% CI 1.469–2.440) for MAFLD. Zong: workday sleep after midnight OR 1.86 (95% CI 1.43–2.42) for MASLD and was also associated with F ≥ 1 and F ≥ 2 fibrosis
Xing et al., 2024^40^	China	Cross-sectional	Adults attending medical-examination centres in the Beijing–Tianjin–Hebei region	39,471	Sleep timing	Sleep midpoint: early before 02:00, intermediate 02:00–02:30, and late after 02:30	Early sleep timing	Prevalent NAFLD	Ultrasonography	Intermediate timing OR 1.15 (95% CI 1.05–1.26); late timing OR 1.08 (95% CI 1.00–1.16). Intermediate/late timing with 7–<8 h sleep OR 1.20 (95% CI 1.04–1.39)

ALT, alanine aminotransferase; CI, confidence interval; DSI, Dallas Steatosis Index; FLI, fatty liver index; FibroScan, vibration-controlled transient elastography device; HR, hazard ratio; HSI, hepatic steatosis index; ICD-10, International Classification of Diseases, 10th Revision; ION, index of NASH; IRR, incidence rate ratio; LFE, liver fat equation; MAFLD, metabolic dysfunction-associated fatty liver disease; MASLD, metabolic dysfunction-associated steatotic liver disease; MRI-PDFF, magnetic resonance imaging proton density fat fraction; NAFLD, nonalcoholic fatty liver disease; NHANES, National Health and Nutrition Examination Survey; OR, odds ratio; USFLI, United States Fatty Liver Index; VCTE, vibration-controlled transient elastography. **Note:** Each study was assigned to a single primary exposure domain for compact tabular presentation, while multidomain findings were retained in the narrative synthesis. Weng et al. and Zong et al.^11^^,^^12^ used overlapping NHANES cycles and were treated as one underlying evidence source rather than independent replication. Tosoratto et al.^38^ assessed surrogate steatosis and fibrosis-risk indices rather than imaging- or biopsy-confirmed MASLD. The mixed-aetiology cirrhosis studies used to contextualize reverse causality were not included in this primary study-characteristics table.

### Characteristics of included studies

Twenty-seven reports corresponding to 26 distinct studies were retained. Of these, 25 reports represented 24 independent MASLD-related evidence sources included in the primary synthesis, because the overlapping Weng et al. and Zong et al. NHANES reports were treated as one underlying evidence source. Two additional reports represented two mixed-aetiology cirrhosis studies considered separately to contextualize reverse causality.

Shift work represented the largest exposure domain, followed by chronotype, objective rhythm or light-related measures, sleep timing, sleep regularity, and circadian-misalignment constructs, and meal timing. Liver outcomes included prevalent and incident NAFLD, MAFLD, or MASLD; moderate-to-severe steatosis; significant and advanced fibrosis; incident cirrhosis among participants with baseline NAFLD; and quantitative liver-fat phenotypes. Outcome ascertainment included ultrasonography, VCTE or FibroScan, biopsy, MRI-based liver-fat assessment, administrative coding, and disease-oriented steatosis or fibrosis indices. Considerable heterogeneity in exposures, comparator categories, outcome definitions, ascertainment methods, adjustment models, and reported effect measures precluded clinically meaningful statistical pooling and supported a descriptive.

### Risk of bias assessment

Risk of bias was assessed for all 24 independent MASLD-related evidence sources using design-appropriate tools. Eighteen cross-sectional studies or analytic components were evaluated with AXIS: 16 were judged at moderate risk and two, Chatur et al. and Kani et al. at high risk. None was judged at low risk. Item-level assessments are presented in Supplementary Table S5, with recurrent methodological concerns summarized in Supplementary Table S5A.

Cross-sectional studies generally reported objectives, designs, target populations, measurements, statistical methods, and limitations adequately. Recurrent concerns were lack of formal sample-size justification, limited representativeness, inadequate non-response information, self-reported timing exposures, indirect liver outcomes, and residual confounding. AXIS review-level judgements were moderate for most studies and high for two (Supplementary Tables S5 and S5A).

Chatur et al. was judged at high risk because of its small selected occupational sample, absence of an external daytime comparator, and limited reporting of representativeness and non-response. Kani et al. was judged at high risk because of its small selected biopsy-based cohort and similar limitations in sampling and non-response assessment. Neither study was used as the sole basis for a domain-level conclusion.

Seven cohort analyses were assessed using the Newcastle–Ottawa Scale and received low overall risk-of-bias judgements, with scores of 8/9-9/9. Strengths included clearly defined cohorts, appropriate exposure ascertainment, confirmation that incident outcomes were absent at baseline, and adequate follow-up. Remaining limitations included self-reported exposures, residual confounding, indirect baseline NAFLD identification in Qin et al., and administrative ascertainment of incident cirrhosis. The single case-control study, Schaeffer et al., received a moderate NOS judgement of 7/9. It used biopsy-confirmed MASLD, clearly defined controls, and consistent circadian assessment, but its small selected clinical sample and limited comparability adjustment restricted generalizability.

Overall, prospective evidence provided stronger support for temporal interpretation, whereas the larger cross-sectional evidence base was limited by representativeness, non-response, residual confounding, and inability to establish temporality. These limitations informed the narrative synthesis and domain-level certainty assessments rather than being treated as a numerical quality score.

### Certainty of evidence

Adapted GRADE certainty ranged from very low to low (Supplementary Table S8). Shift work, chronotype, objective rest-activity/light/circadian biomarkers, and meal timing were rated low; sleep timing, sleep regularity, and circadian-misalignment constructs were very low because evidence was sparse, cross-sectional, partly overlapping, and based largely on self-report. No domain met criteria for moderate or high certainty.

### Narrative synthesis of findings

**Fig. 2:**
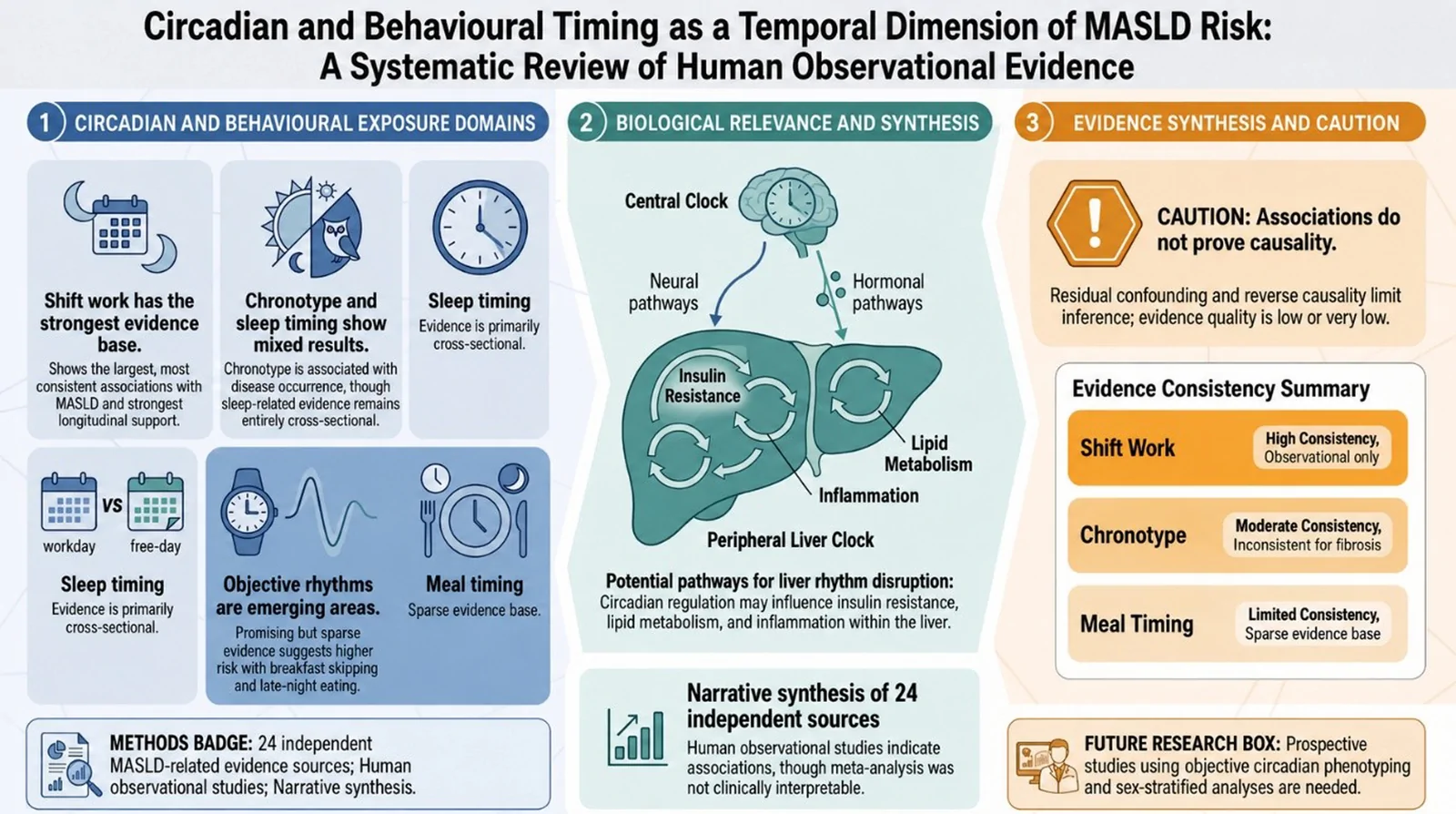
**Circadian and behavioural timing as a temporal dimension of MASLD risk: a systematic review of human observational evidence.** Schematic summary of human observational evidence linking circadian and behavioural timing exposures with metabolic dysfunction-associated steatotic liver disease (MASLD). (1) Circadian and behavioural exposure domains: Shift work has the strongest and most consistent association with MASLD and greater longitudinal support, whereas evidence for chronotype, sleep timing, objective circadian rhythms, and meal timing is more limited or predominantly cross-sectional. (2) Biological relevance and synthesis: Circadian disruption may influence hepatic insulin resistance, lipid metabolism, inflammation, and the peripheral liver clock through neural and hormonal pathways originating from the central clock. Narrative synthesis of 24 independent sources suggests biologically plausible associations, although substantial heterogeneity limits causal interpretation. (3) Evidence synthesis and caution: Observed associations do not establish causality because of residual confounding, reverse causation, and generally low-to-very-low evidence quality. Evidence is most consistent for shift work, moderate but inconsistent for chronotype, and limited for meal timing. Prospective studies incorporating objective circadian phenotyping and sex-stratified analyses are needed.

**Fig. 3:**
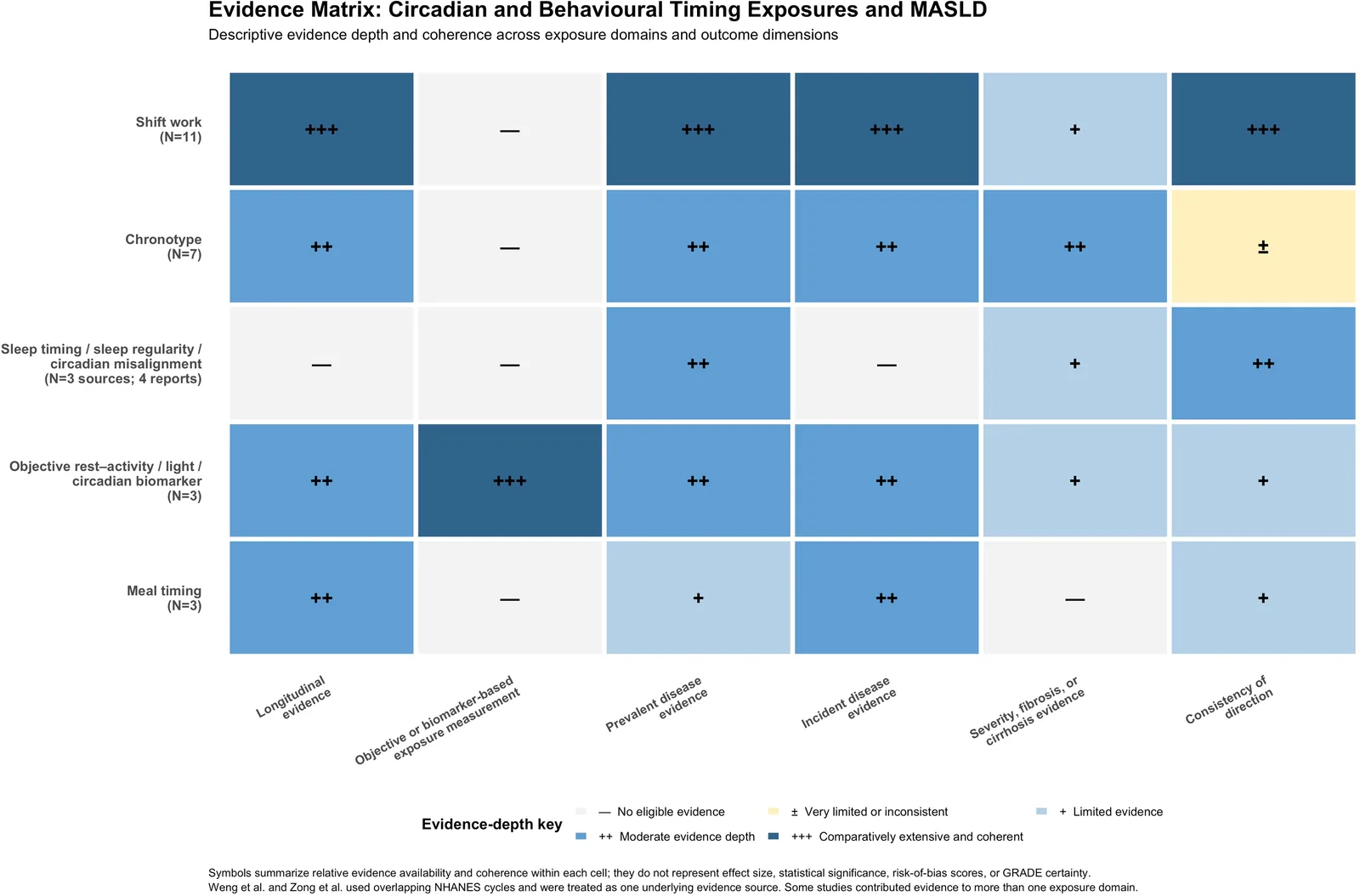
**Domain-level descriptive evidence matrix for circadian and behavioural timing exposures and MASLD-related outcomes.** Symbols indicate the relative depth and coherence of evidence within each domain: —, no eligible evidence; ±, very limited or inconsistent evidence; +, limited evidence; ++, moderate evidence depth; and +++, comparatively extensive and coherent evidence. Ratings considered the number and design of contributing studies, replication across independent populations, outcome coverage, consistency of findings, and major methodological limitations. Symbols do not represent effect magnitude, statistical significance, risk-of-bias scores, or GRADE certainty. Reports from overlapping populations were treated as one underlying evidence source.

The included studies were synthesized narratively according to five circadian and behavioural timing domains: shift-work-related exposures; chronotype; sleep timing, sleep regularity, and study-defined circadian-misalignment constructs beyond sleep duration; objective rest–activity, light, and circadian biomarker measures; and meal-timing-related exposures. Several studies contributed to more than one domain; therefore, domain-specific study counts should not be interpreted as mutually exclusive. Because exposure definitions, liver outcome ascertainment methods, comparator groups, and covariate-adjustment strategies varied substantially across studies, results are synthesized narratively with emphasis on the direction, magnitude, and consistency of adjusted associations rather than on a single pooled estimate (Fig. 2). A domain-level descriptive evidence matrix summarizing study-design depth, measurement characteristics, liver-outcome coverage, and consistency across the five circadian exposure domains is presented in Fig. 3. Symbols in the matrix indicate the relative availability and coherence of evidence within each column and do not represent pooled effect size, statistical significance, or certainty of evidence. The corresponding study counts, outcomes, limitations, and narrative interpretations are presented in Table 2.

**Table 2:** Evidence synthesis by exposure domain.

Exposure domain	No. of contributing studies	Study designs represented	Main liver outcomes represented	Overall direction of association	Evidence for incident disease	Evidence for severity/fibrosis	Main methodological limitations	Overall interpretive summary
Shift work-related exposures	11	prospective cohort; longitudinal cohort; cross-sectional	prevalent and incident NAFLD/MAFLD/MASLD; moderate-to-severe steatosis	predominantly positive	strong	limited	Heterogeneous definitions; sex-skewed cohorts; variable outcomes; residual adiposity-related confounding.	Largest and most consistent evidence base, with the strongest longitudinal support; associations may reflect combined circadian, sleep, dietary, and occupational effects.
Chronotype	7	prospective and longitudinal cohort studies; cross-sectional studies	prevalent NAFLD; incident MAFLD; significant and advanced fibrosis; incident cirrhosis	supportive for disease occurrence; mixed for advanced outcomes	present for incident MAFLD and cirrhosis	inconsistent	Mostly self-reported exposure; selected populations; imprecise fibrosis estimates; inconsistent advanced-outcome findings.	supportive evidence for disease occurrence, but findings for fibrosis and cirrhosis were inconsistent; effects of schedule mismatch remain unclear.
Sleep timing, sleep regularity, and circadian-misalignment constructs	3 independent sources across 4 reports	cross-sectional analyses	prevalent NAFLD/MAFLD/MASLD and VCTE-defined liver fibrosis	generally positive but limited	none	limited; reported in one overlapping NHANES analysis	cross-sectional designs; self-reported timing; overlapping NHANES samples; residual confounding; no prospective evidence	possible associations with prevalent MASLD and fibrosis, but evidence is cross-sectional and of very low certainty
Objective rest-activity, light, and circadian biomarker measures	3	prospective cohort; cross-sectional analysis; case-control clinical study	prevalent and incident MASLD and biopsy-characterized MASLD phenotypes	directionally supportive but heterogeneous	present	limited	heterogeneous metrics; and outcomes; imperfect light proxies; small clinical samples; limited melatonin phase	Objective rhythm and light findings were generally supportive; melatonin-phase evidence was limited to one small case-control study
Meal timing-related exposures	3	prospective cohort and cross-sectional	incident MASLD; prevalent NAFLD	positive but limited	present	not clearly demonstrated	self-reported meal timing; limited adjustment for diet composition; sparse prospective evidence	promising but comparatively underdeveloped chrononutritional domain

**Note:** Domain counts are not mutually exclusive because some studies contributed to more than one domain. Table summarizes evidence volume, design, measurement, outcomes, consistency, limitations, and GRADE certainty; it does not rank causal importance or effect magnitude.

### Shift work-related exposures and steatotic liver disease outcomes

Eleven independent evidence sources evaluated shift work in relation to MASLD-related outcomes, making it the largest exposure domain. Shift work was interpreted as an externally imposed, multidimensional occupational exposure rather than an individual circadian characteristic because night and rotating schedules may simultaneously affect sleep, light exposure, food timing, physical activity, occupational stress, and social routines. Studies included occupational and population-based cohorts of steelworkers, railway and subway employees, automobile-manufacturing workers, nurses, security personnel, Spanish workers across employment sectors, and UK Biobank participants. Outcomes included prevalent and incident NAFLD, MAFLD, or MASLD, moderate-to-severe steatosis, and surrogate steatosis or fibrosis-risk indices.

The strongest temporal evidence came from longitudinal studies.^24^^,^^27^^,^^34^ Huang et al. (2023)^24^ reported that usual or permanent night shift work was associated with incident NAFLD in UK Biobank participants, with higher estimates among those with longer lifetime exposure. Xu et al. (2023)^34^ similarly reported higher incident NAFLD risk among frequent, but not occasional, shift workers. Lee and Lee (2024)^27^ identified an age-specific association among workers in their twenties identified an age-specific association among workers in their twenties, although this finding requires confirmation in additional cohorts.

Cross-sectional occupational studies were generally directionally consistent, reporting higher prevalence or risk with shift-work status, greater monthly frequency, longer duration, or cumulative night-shift burden. Tosoratto et al. (2025)^39^ found associations with several non-invasive steatosis and fibrosis-risk indices in a large Spanish occupational population, although these indices incorporated anthropometric, metabolic, or biochemical variables and did not represent imaging- or biopsy-confirmed MASLD.

Overall, shift work showed the most consistent associations and contained the largest concentration of longitudinal evidence. This does not establish that shift work is a stronger biological circadian determinant than chronotype. Rather, it represents a more intensive composite exposure whose effects cannot be separated confidently from sleep restriction, nocturnal light, nighttime eating, occupational stress, job characteristics, socioeconomic conditions, and correlated behaviours.

### Chronotype and fatty liver disease occurrence or severity

Seven independent evidence sources evaluated chronotype in relation to MASLD occurrence or severity. Chronotype is a relatively stable preference for earlier or later sleep and activity rather than an externally imposed exposure. Evening chronotype should therefore not be considered circadian disruption in itself; any adverse association may depend on conflict between preferred timing and occupational, social, light-dark, sleep, or meal schedules.

Fu et al. provided longitudinal evidence that evening chronotype was associated with incident MAFLD. Cross-sectional findings from Hu et al.,^23^ Maidstone et al.,^28^ and Vetrani et al.,^31^ generally supported associations with prevalent disease or indirect severity measures, although populations and outcome definitions differed. Findings for advanced disease were inconsistent. Castelnuovo et al. reported independent associations of intermediate or late chronotype with FibroScan-defined significant and advanced fibrosis among 126 adults with NAFLD, but estimates were highly imprecise. Kani et al. found no significant association with biopsy-defined advanced fibrosis.

Qin et al.,^38^ prospectively examined incident cirrhosis among 112,196 UK Biobank participants with baseline NAFLD. Morning chronotype was not independently associated with cirrhosis. Although a favourable composite sleep score was associated with lower risk, it also included sleep duration, insomnia, daytime dozing, and snoring and was therefore not interpreted as a chronotype-specific effect. Overall, chronotype evidence was more supportive for disease occurrence than for advanced fibrosis or cirrhosis. The studies did not establish whether associations reflected endogenous timing, behavioural correlates of later timing, or misalignment between chronotype and externally imposed schedules. Chronotype-by-work-schedule interactions were not adequately evaluated.

### Sleep timing, sleep regularity, and circadian misalignment constructs beyond sleep duration

Three independent evidence sources, represented by four reports, evaluated behavioural sleep timing, sleep regularity, or composite circadian-misalignment measures beyond sleep duration. Weng et al. examined a composite of mistimed, late, and irregular sleep, which was associated with prevalent MAFLD. Zong et al.,^13^ using an expanded but overlapping NHANES population, reported that workday sleep after midnight was associated with MASLD and mild and significant fibrosis. Because the two analyses shared NHANES cycles, they were treated as one evidence source rather than independent replication.

Chen et al.,^20^ found that weekend catch-up sleep exceeding 1 h was associated with prevalent MASLD, although the association was sensitive to adiposity adjustment. Xing et al.,^40^ reported higher odds of ultrasound-defined NAFLD among participants with intermediate or late sleep midpoint compared with early timing. Delayed timing remained associated among those sleeping 7-<8 h, although the continuously modelled sleep-midpoint association became null after full adjustment.

These findings suggest that later timing and greater schedule variability may provide information beyond sleep duration alone. However, the constructs were heterogeneous and should not be interpreted as equivalent measures of chronotype or endogenous circadian phase. All studies were cross-sectional, used self-reported timing measures, and could not establish temporality.

### Objective rhythm, light, and circadian biomarker measures in relation to MASLD

Three independent evidence sources evaluated objective rest-activity rhythms, light exposure, or melatonin phase. Wu et al. provided the strongest evidence, reporting lower incident MASLD risk with higher rest-activity relative amplitude and greater risk with higher nocturnal light exposure. Gu et al.,^22^ provided supportive cross-sectional evidence linking more favourable rest-activity and feeding-fasting rhythm metrics with lower odds of suspected NAFLD.

Schaeffer et al.,^32^ assessed a small clinical sample using actigraphy and serial salivary melatonin measurements. Patients with biopsy-confirmed MASLD showed greater nocturnal wakefulness, longer wakefulness after sleep onset, and lower sleep efficiency, but no clear shift in dim-light melatonin onset.

This domain used more direct circadian measures than most questionnaire-based studies, but interpretation was limited by heterogeneous metrics, wrist-measured light as an imperfect proxy for biologically effective ocular exposure, differing liver outcomes, and the small selected clinical sample.

### Meal timing-related exposures and fatty liver outcomes

Meal-timing evidence was limited but generally consistent. In 32,030 adults followed for a median 6.34 years, Shi et al. (2025)^30^ found higher incident MASLD with never eating breakfast (HR 1.35, 95% CI 1.25–1.45), regular late-night snacking (HR 1.15, 95% CI 1.08–1.21), and especially both behaviours (HR 1.52, 95% CI 1.30–1.77).

Gu et al. (2023)^22^ found that a more favourable feeding-rhythm score was associated with lower NAFLD odds (OR 0.74, 95% CI 0.58–0.95). Kani et al. (2023)^25^ evaluated night eating in biopsy-confirmed MASLD but found no clear association with severity. Interpretation was limited by self-reported meal timing and incomplete adjustment for total energy intake, macronutrient composition, and broader dietary patterns. Men were also overrepresented in the largest cohort. Overall, breakfast skipping and late-night snacking were associated with higher MASLD risk, but this domain remains underdeveloped and requires further prospective research with detailed chrononutritional assessment.

### Melatonin phase assessment within the objective rhythm and light domain

MASLD-specific evidence on endogenous melatonin was limited to one small Swiss case-control study using serial salivary sampling and dim-light melatonin-onset estimation. Schaeffer et al. found no clear difference in melatonin-onset timing or salivary profiles between patients with biopsy-confirmed MASLD and healthy controls. The MASLD group nevertheless showed greater nocturnal wakefulness, longer wakefulness after sleep onset, and lower sleep efficiency.

These findings suggest that sleep fragmentation may occur without detectable melatonin-phase displacement. However, the study was small, non-longitudinal, and did not assess melatonin as a predictor of fibrosis progression. It therefore cannot determine whether sleep disturbance preceded MASLD, resulted from liver disease, or reflected other clinical factors.

Melatonin must be interpreted in relation to sampling time, light exposure, biological matrix, assay validity, and the temporal profile. A single concentration cannot identify circadian phase, amplitude, total secretion, or rhythmicity; repeated timed sampling, dim-light melatonin onset, or sufficiently dense 24-h profiling is required.

### Circadian abnormalities in established cirrhosis

Two mixed-aetiology cirrhosis studies were examined separately to assess whether advanced liver disease may itself alter circadian physiology. They were not included among the 24 independent MASLD-related evidence sources.

Córdoba et al.,^41^ reported frequent sleep disturbance, delayed bedtime and wake time, and greater evening preference among patients with cirrhosis. Montagnese et al.^42^ assessed 20 patients and nine controls using controlled 24-h hormonal profiling, urinary 6-sulfatoxymelatonin, nocturnal light-suppression testing, actigraphy, and sleep diaries. Patients showed delayed melatonin and cortisol peaks and reduced melatonin suppression after nocturnal light, while mean 24-h melatonin clearance did not differ significantly.

These studies support altered circadian phase, light responsiveness, and sleep-wake organization in established cirrhosis, but not a simple relationship between absolute melatonin concentration and fibrosis severity. They were interpreted as evidence of possible reverse causality and were not used to infer that melatonin abnormalities caused fibrosis or preceded MASLD progression.

### Cross-domain synthesis of the clinical evidence

Across 24 independent MASLD-related evidence sources, five circadian and behavioural timing domains were identified. Cross-domain comparisons considered evidence volume, study design, exposure measurement, outcome coverage, replication, consistency, and methodological limitations. Matrix ratings were therefore interpreted as descriptive summaries rather than numerical rankings of causal importance or treatment relevance.

Shift work contributed 11 evidence sources and the largest concentration of longitudinal studies. Chronotype contributed seven sources and included evidence for disease occurrence, fibrosis, and cirrhosis, although advanced-disease findings were inconsistent. Sleep timing, sleep regularity, and circadian-misalignment constructs were represented by three independent sources across four reports. Objective rest-activity, light, and circadian biomarker measures contributed three sources, and meal timing contributed three. Overlapping reports were linked and not counted as independent replication.

Occupational shift work provided the largest and most internally consistent evidence base. However, it represents a multidimensional exposure that may simultaneously alter sleep, light exposure, meal timing, physical activity, occupational stress, and social routines. Chronotype, by contrast, is an individual timing phenotype that may act partly as a susceptibility factor. These domains were therefore interpreted separately rather than ranked directly.

Considerable heterogeneity remained in both exposure and outcome definitions. Exposures included night work, cumulative shift burden, evening chronotype, weekend catch-up sleep, late or irregular sleep, meal-timing disruption, reduced rest-activity amplitude, and nocturnal light exposure. Liver outcomes ranged from biopsy-confirmed MASLD/MASH and elastography-defined fibrosis to ultrasonography, MRI-derived liver fat, ICD-coded disease, and indirect steatosis or fibrosis indices. Adjustment strategies also varied, particularly for adiposity and metabolic factors, which may function as confounders, mediators, or both.

Prospective evidence was available for shift work, chronotype, meal timing, and objective rhythm or light measures, supporting temporal interpretation but not causality. In contrast, all studies of sleep timing, sleep regularity, and circadian-misalignment constructs were cross-sectional, and the only eligible melatonin-phase study used a case-control design. Reverse causality therefore remains plausible, particularly because established liver disease may itself contribute to sleep fragmentation, fatigue, altered activity rhythms, and circadian disturbance.

**Fig. 4:**
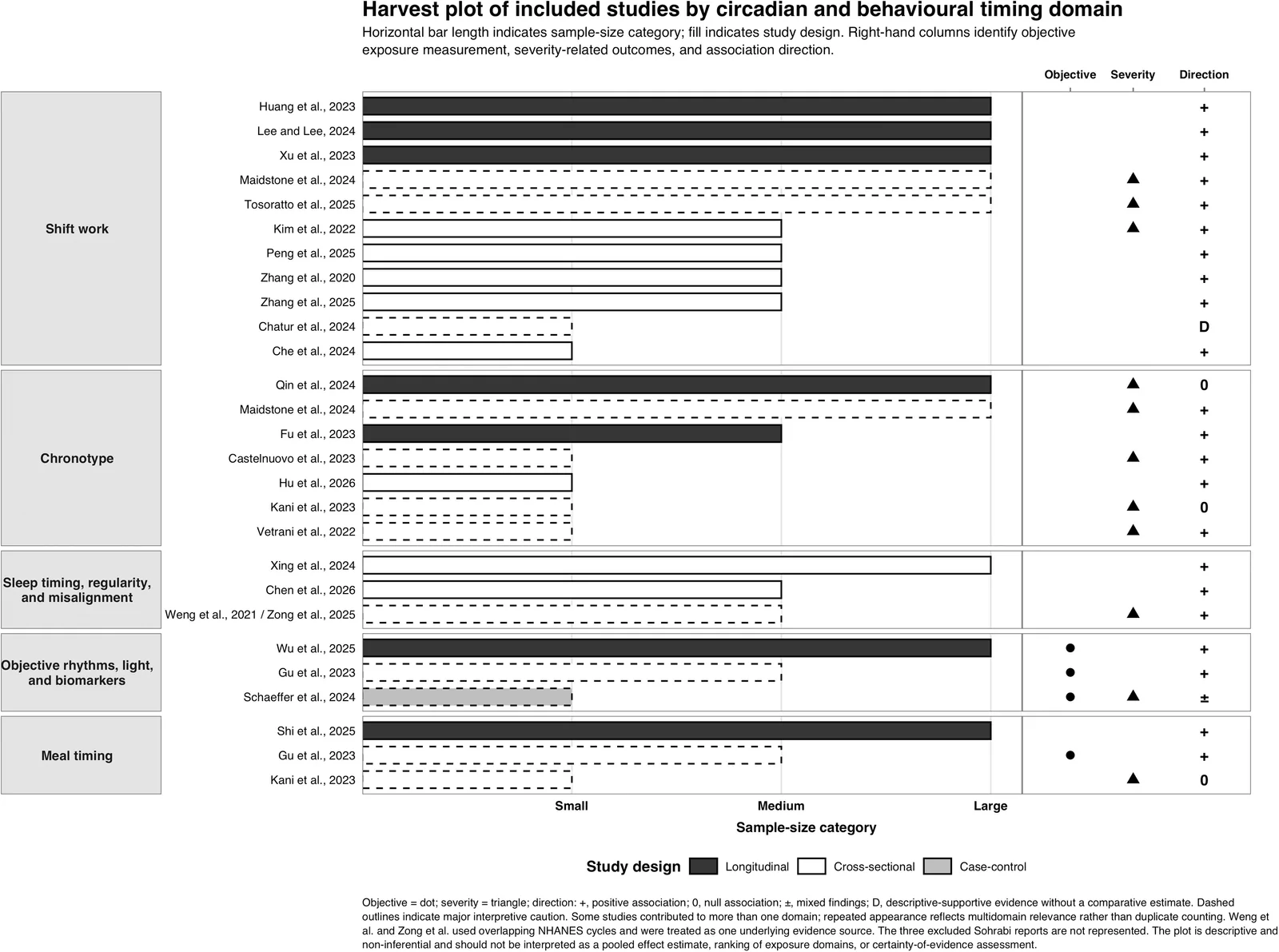
**Harvest plot of included studies by circadian and behavioural timing domain.** Each horizontal bar represents an included study or underlying evidence source. Bar length indicates sample-size category: small (<1000 participants), medium (1000–9999), or large (≥10,000), and is not proportional to effect magnitude or statistical weight. Bar fill identifies study design as longitudinal, cross-sectional, or case-control. Dots indicate objective or biomarker-based exposure measurement, and triangles indicate severity-, fibrosis-, cirrhosis-, biopsy-, or liver-fat-fraction-related outcomes. Association direction is represented by + for a positive association, 0 for a null association, ± for mixed findings, and D for descriptive-supportive evidence without a comparative estimate. Dashed outlines indicate major interpretive caution but do not constitute a separate formal risk-of-bias category. Repeated appearance across domains reflects multidomain relevance rather than duplicate counting. Weng et al. and Zong et al. were treated as one underlying NHANES evidence source. The plot is descriptive and non-inferential and should not be interpreted as a pooled effect estimate, ranking of exposure domains, or certainty-of-evidence assessment.

Overall, the evidence was strongest for occupational shift work, while sleep timing and regularity may provide information beyond sleep duration and meal timing represents a promising but underdeveloped chrononutritional pathway. The synthesis maps the direction and consistency of associations rather than demonstrating independent causal effects. Statistical pooling was not clinically appropriate because exposure definitions, comparators, outcome ascertainment, and analytical models differed substantially. The study-level distribution of association direction, design, sample size, and methodological characteristics is summarized in Fig. 4.

## Discussion

This systematic review synthesized human observational evidence on circadian disruption-related exposures and MASLD-related liver outcomes. Across 24 independent evidence sources, shift work was the most consistently supported domain and contributed the greatest concentration of longitudinal evidence. Chronotype and sleep-timing studies extended the evidence beyond disease occurrence to significant fibrosis, advanced fibrosis, and incident cirrhosis, although advanced-disease findings were inconsistent. Intermediate or late chronotype was associated with fibrosis in one small clinical cohort, whereas morning chronotype was not independently associated with incident cirrhosis in a large prospective NAFLD cohort. Later or irregular sleep timing was associated with prevalent disease and, in one overlapping NHANES analysis, liver fibrosis. These findings suggest a possible temporal dimension of MASLD risk but do not establish an independent causal effect.

MASLD is a multifactorial condition shaped by adiposity, insulin resistance, diet, physical activity, socioeconomic context, occupational conditions, alcohol exposure, and sleep health.^1^^,^^2^ Circadian disruption may therefore be better understood as a timing-related framework that interacts with established metabolic pathways rather than as a single isolated cause. The biological rationale remains plausible because the hepatic peripheral clock regulates glucose handling, lipid metabolism, bile acid pathways, mitochondrial activity, inflammation, and detoxification.^4^, ^5^, ^6^ Human laboratory studies also show that circadian misalignment adversely affects insulin sensitivity, glucose regulation, cortisol rhythms, leptin signalling, blood pressure, and inflammatory markers.^7^^,^^8^ These mechanisms are compatible with the observed associations but do not prove liver-specific causality.

Chronotype reflects a relatively stable preference for earlier or later sleep and activity timing. Although biologically influenced, it also varies with age, light exposure, and social context. Evening chronotype is therefore not inherently pathological and should not be treated as synonymous with circadian disruption.

Shift work is an externally imposed occupational exposure that may require wakefulness, light exposure, activity, and food intake during the biological night and sleep during the biological day. It also encompasses sleep restriction, schedule variability, occupational demands, psychosocial stress, socioeconomic conditions, and workplace behaviours. Its stronger and more consistent associations may therefore reflect both greater circadian challenge and additional non-circadian occupational exposures.

The clinically relevant factor may be alignment between chronotype and required schedules rather than either characteristic alone. Evening-oriented individuals may tolerate some later schedules better than morning-oriented individuals, whereas early schedules may be more disruptive for later chronotypes. Adaptation to night work may also vary according to chronotype, rotation pattern, and sleep opportunity. However, the included studies rarely assessed chronotype and work schedule jointly, preventing firm conclusions regarding chronotype-specific vulnerability.

Shift work was the most consistently supported domain and included large occupational and population-based cohorts with the clearest prospective evidence. However, it may combine circadian misalignment, sleep restriction, nocturnal light exposure, nighttime eating, psychosocial stress, occupational noise, and reduced opportunity for healthy behaviours. Associations may therefore reflect both timing disruption and correlated occupational or lifestyle factors. Attenuation after adiposity adjustment in some studies further suggests that BMI and metabolic status may act as confounders, mediators, or both.

Biological sex was inconsistently represented and analysed. Shift-work studies included male-only or male-predominant industrial cohorts and an all-female nursing cohort. Although many mixed-sex studies adjusted for sex, few reported sex-stratified estimates or formally tested exposure-by-sex interactions. Occasional subgroup differences were difficult to interpret because of unequal representation and limited statistical power. No included study directly assessed gender identity, gender roles, or gender-related social determinants. The evidence therefore supports only limited sex-specific interpretation and no conclusions regarding gender differences (Supplementary Table S9).

Chronotype, observed sleep timing, and sleep regularity are related but distinct. Chronotype reflects preferred timing, whereas bedtime, wake time, sleep midpoint, and day-to-day variability describe realized schedules influenced by work, family, and social obligations. Social jetlag reflects differences between workday and free-day timing, whereas circadian misalignment refers more specifically to discordance between endogenous timing and imposed behavioural or environmental schedules.

An evening chronotype does not necessarily imply an irregular sleep schedule, and irregular timing does not constitute an “irregular chronotype.” Most sleep-timing and regularity studies were cross-sectional and relied on self-report. Prospective studies using actigraphy, repeated sleep diaries, validated chronotype instruments, and physiological phase markers are needed to determine whether chronotype, late sleep timing, schedule variability, and circadian misalignment have independent relationships with MASLD.

Advanced liver disease also creates important reverse-causality and measurement concerns. Controlled cirrhosis studies have reported delayed sleep-wake timing, delayed melatonin and cortisol phase, altered melatonin responses to nocturnal light, and misalignment between biological and behavioural timing (Supplementary Table S10). These findings concern the timing and regulation of circadian signals rather than a uniform increase or decrease in absolute melatonin concentration.

An isolated melatonin value cannot therefore be interpreted as evidence of circadian phase disturbance or fibrosis progression, particularly when sampling time, light conditions, assay characteristics, and disease severity are incompletely described. Cross-sectional associations cannot determine whether circadian abnormalities preceded fibrosis, resulted from advanced liver disease, or reflected measurement conditions. No direct mechanistic pathway linking circulating melatonin concentrations with fibrosis progression can be inferred from the available clinical evidence.

Meal-timing evidence was promising but sparse. The largest prospective study found that breakfast skipping and late-night snacking were associated with incident MASLD, particularly when both occurred together.^30^ Feeding–fasting cycles are major entraining signals for peripheral clocks, especially the liver clock. Mechanistic studies indicate that feeding time can shift hepatic transcriptional rhythms and influence CLOCK/BMAL1, PER-family genes, NAD+ -dependent SIRT1 signalling, glucose handling, lipid metabolism, and inflammatory regulation.^5^^,^^43^, ^44^, ^45^ However, meal timing may also reflect total energy intake, diet quality, work schedules, socioeconomic constraints, sleep timing, alcohol timing, or broader lifestyle irregularity, and the current evidence does not establish an independent causal effect.

The findings do not establish that circadian disruption independently causes MASLD. Shift work showed the most consistent associations but is a complex occupational exposure encompassing sleep restriction, nocturnal light, nighttime eating, schedule rotation, occupational stress, physical activity, job characteristics, and socioeconomic conditions. Chronotype is a trait-like timing phenotype and should not be considered an exposure of equivalent type or intensity. Evidence was insufficient to determine whether chronotype independently influences MASLD risk or modifies the effects of imposed work schedules.

Interpretation was further limited by heterogeneous exposure definitions, cross-sectional designs, residual confounding, exposure misclassification, reverse causality, and adjustment for variables that may act as confounders or mediators. The findings therefore identify recurrent observational associations and research priorities rather than causal relationships.

Intervention studies address the distinct question of whether modifying light exposure, sleep or work schedules, meal timing, or melatonin signalling improves MASLD-related outcomes. Beneficial effects would support modifiability but would not confirm that naturally occurring circadian disruption caused MASLD, while null findings could reflect inadequate intensity, adherence, duration, or timing. Intervention evidence should therefore remain separate from observational exposure-outcome evidence.

The adapted GRADE assessment supported cautious interpretation.^16^ Certainty was low for shift work, chronotype, objective rest-activity, light and circadian biomarker measures, and meal timing, and very low for sleep timing, sleep regularity, and circadian-misalignment constructs. The latter domain comprised only three independent cross-sectional evidence sources across four reports, included overlapping NHANES cycles and self-reported exposures, and lacked prospective evidence.

Current evidence does not support circadian screening as a validated MASLD risk-stratification tool or justify circadian-targeted treatment recommendations. Timing-related exposures may nevertheless be considered within broader lifestyle assessment, including shift-work patterns, chronotype, workday-free-day sleep differences, breakfast skipping, late-night eating, and the timing of caloric and alcohol intake. These remain hypothesis-informed prompts rather than validated clinical tools.

A dedicated synthesis for MetALD was not possible, representing an important gap under the updated steatotic liver disease nomenclature. Future studies should distinguish MASLD from MetALD and assess alcohol quantity, timing, and pattern alongside sleep, light, meal timing, and occupational exposures.

Strengths of this review include its domain-based framework, separation of circadian timing from general sleep traits, inclusion of advanced fibrosis and cirrhosis outcomes, explicit handling of overlapping reports, design-appropriate risk-of-bias assessment, domain-level certainty assessment, and consideration of sex representation, temporality, confounding, and reverse causality.

Limitations included heterogeneous exposures and outcomes, frequent self-report, predominantly cross-sectional evidence, variable adjustment for adiposity and metabolic factors, unequal male and female representation, inconsistent sex-specific analyses, and absence of gender-related measures. Statistical pooling was not undertaken because clinically coherent exposure–outcome groups were too sparse and heterogeneous. Melatonin evidence was especially limited: three overlapping reports were excluded because specimen timing and assay identification were inadequately reported, leaving one small case-control study using serial salivary sampling and dim-light melatonin-onset estimation. Evidence for MetALD was also sparse, and certainty remained low or very low across all domains.

Circadian and behavioural timing exposures were recurrently associated with MASLD-related outcomes in human observational studies, with the most consistent and longitudinally supported evidence observed for shift work. Evidence for chronotype, sleep timing, objective rest–activity and light-related measures, and meal timing was less developed and remained limited by heterogeneous exposure definitions, variable liver-outcome ascertainment, residual confounding, and frequent cross-sectional designs. Findings for significant fibrosis, advanced fibrosis, and incident cirrhosis were inconsistent, and evidence from established cirrhosis demonstrated that advanced liver disease can itself alter circadian physiology. The results therefore describe the possible direction and consistency of associations across heterogeneous observational studies and do not establish an independent causal relationship. Prospective studies using objective circadian phenotyping, repeated exposure assessment, robust confounder control, and standardized imaging or histological outcomes are required to clarify the temporal relationship between circadian disruption and MASLD.

## Contributors

AS, and VS conceptualized and designed the study. AS and VS curated the data. AS, RARA, GSD, and VS accessed and verified the underlying study data. AS and VS contributed to data interpretation. AS did the formal data analysis. AS, RARA, GSD, and VS contributed to the methodology. RARA and GSD administered the project. VS supervised the project. AS, and VS wrote the original draft, and all authors contributed to data interpretation and writing, reviewing, and editing this manuscript. All authors read and approved the final version of the manuscript.

## Data sharing statement

Data supporting the findings are available in the Supplementary Material and from the corresponding author upon request.

## Declaration of interests

AS, RARA, GSD, and VS declare no competing interests.

## References

[1] Rinella M.E., Lazarus J.V., Ratziu V. (2023). A multisociety Delphi consensus statement on new fatty liver disease nomenclature. J Hepatol.

[2] Tacke F., Horn P., Wai-Sun Wong V. (2024). EASL–EASD–EASO Clinical Practice Guidelines on the management of metabolic dysfunction-associated steatotic liver disease (MASLD). J Hepatol.

[3] Riazi K., Azhari H., Charette J.H. (2022). The prevalence and incidence of NAFLD worldwide: a systematic review and meta-analysis. Lancet Gastroenterol Hepatol.

[4] Bass J., Takahashi J.S. (2010). Circadian integration of metabolism and energetics. Science.

[5] Panda S. (2016). Circadian physiology of metabolism. Science.

[6] Mukherji A., Bailey S.M., Staels B., Baumert T.F. (2019). The circadian clock and liver function in health and disease. J Hepatol.

[7] Morris C.J., Purvis T.E., Hu K., Scheer F.A.J.L. (2016). Circadian misalignment increases cardiovascular disease risk factors in humans. Proc Natl Acad Sci USA.

[8] Scheer F.A.J.L., Hilton M.F., Mantzoros C.S., Shea S.A. (2009). Adverse metabolic and cardiovascular consequences of circadian misalignment. Proc Natl Acad Sci USA.

[9] Page M.J., McKenzie J.E., Bossuyt P.M. (2021). The PRISMA 2020 statement: an updated guideline for reporting systematic reviews. BMJ.

[10] Schiavo J.H. (2019). PROSPERO: an international register of systematic review protocols. Med Ref Serv Q.

[11] Ouzzani M., Hammady H., Fedorowicz Z., Elmagarmid A. (2016). Rayyan—a web and mobile app for systematic reviews. Syst Rev.

[12] Weng Z., Ou W., Huang J. (2021). Circadian misalignment rather than sleep duration is associated with MAFLD: a population-based propensity score-matched study. Nat Sci Sleep.

[13] Zong G., Mao W., Wen M., Cheng X., Liu G. (2025). Association of sleep patterns and disorders with metabolic dysfunction-associated steatotic liver disease and liver fibrosis in contemporary American adults. Ann Hepatol.

[14] Downes M.J., Brennan M.L., Williams H.C., Dean R.S. (2016). Development of a critical appraisal tool to assess the quality of cross-sectional studies (AXIS). BMJ Open.

[15] Gualdi-Russo E., Zaccagni L. (2026). The Newcastle–Ottawa scale for assessing the quality of studies in systematic reviews. Publications.

[16] Higgins J.P.T., Thomas J., Chandler J. (2019).

[17] Guyatt G.H., Oxman A.D., Vist G.E. (2008). GRADE: an emerging consensus on rating quality of evidence and strength of recommendations. BMJ.

[18] Chatur D.K., Pati S.K., Ghate J.R., Nanda R., Sinha M., Kodapi K. (2024). Nonalcoholic fatty liver disease in shift workers and its effect on peripheral nerve conduction: a cross-sectional study. Cureus.

[19] Che Y., Tang R., Zhang H. (2024). Development of a prediction model for predicting the prevalence of nonalcoholic fatty liver disease in Chinese nurses: the first-year follow data of a web-based ambispective cohort study. BMC Gastroenterol.

[20] Chen J., Wang W., He J., Fang X. (2026). Association between weekend catch-up sleep and metabolic dysfunction-associated steatotic liver disease in US adults. Clin Exp Med.

[21] Fu Y., Yu B., Yang B. (2023). Association between chronotype and metabolic-associated fatty liver disease in employed adults: a longitudinal study in Southwestern China. Chronobiol Int.

[22] Gu W., Han T., Sun C. (2023). Association of 24 h behavior rhythm with non-alcoholic fatty liver disease among American adults with overweight/obesity. Nutrients.

[23] Hu M.-J., Dong X.-M., Wang X.-L. (2026). Chronotype, serum metabolome, and nonalcoholic fatty liver disease in middle-aged and older adults: association and potential mediation analyses. Exp Gerontol.

[24] Huang H., Liu Z., Xie J., Xu C. (2023). Association between night shift work and NAFLD: a prospective analysis of 281,280 UK Biobank participants. BMC Public Health.

[25] Sakalli Kani A. (2023). Chronotype preference, sleep quality, and night-eating behaviors in patients with metabolic dysfunction-associated steatotic liver disease: assessing the relationship with disease severity and fibrosis. Hepatol Forum.

[26] Kim K., Lee Y.-J., Kwon S.-C. (2022). Correlation between shift work and non-alcoholic fatty liver disease among male workers in the steel manufacturing company of Korea: a cross-sectional study. Ann Occup Environ Med.

[27] Lee Y., Lee W. (2024). Shift work and non-alcoholic fatty liver disease in young, healthy workers. Sci Rep.

[28] Maidstone R., Rutter M.K., Marjot T., Ray D.W., Baxter M. (2023). Shift work and evening chronotype are associated with hepatic fat fraction and non-alcoholic fatty liver disease in 282,303 UK biobank participants. Endocr Connect.

[29] Peng R., Shi B., Liu J., He Z. (2025). Association of shift work with metabolic dysfunction-associated fatty liver disease among subway workers. Front Public Health.

[30] Shi W., Suo X., Wang Y. (2025). The associations between irregular breakfast and late-night snacking with metabolic dysfunction-associated steatotic liver disease. Clin Nutr.

[31] Vetrani C., Barrea L., Verde L. (2022). Evening chronotype is associated with severe NAFLD in obesity. Int J Obes.

[32] Schaeffer S., Bogdanovic A., Hildebrandt T. (2024). Significant nocturnal wakefulness after sleep onset in metabolic dysfunction–associated steatotic liver disease. Front Netw Physiol.

[33] Wu H., Wang W., Weng B. (2026). Diurnal light exposure and rest-activity rhythms in relation to MASLD: insights from 2 nationwide cohort studies. J Clin Endocrinol Metab.

[34] Xu J., Ni S., Wang Y. (2023). Shift work and nonalcoholic fatty liver disease incidence among Chinese rail workers: a 4-year longitudinal cohort study. Int Arch Occup Environ Health.

[35] Zhang J., Zhang Y., Qiu C.X. (2025). Association of occupational noise exposure and shift work with non-alcoholic fatty liver disease: a cross-sectional study of male workers in the Chinese automobile manufacturing industry. BMJ Open.

[36] Zhang S., Wang Y., Wang Z. (2020). Rotating night shift work and non-alcoholic fatty liver disease among steelworkers in China: a cross-sectional survey. Occup Environ Med.

[37] Castelnuovo G., Perez-Diaz-del-Campo N., Rosso C. (2023). Impact of chronotype and mediterranean diet on the risk of liver fibrosis in patients with non-alcoholic fatty liver disease. Nutrients.

[38] Qin S., Cheng X., Zhang S. (2024). Sleep patterns, genetic susceptibility, and risk of cirrhosis among individuals with nonalcoholic fatty liver disease. Hepatol Int.

[39] Tosoratto J., Tárraga López P.J., López-González Á.A., Busquets-Cortes C., Obrador de Hevia J., Ramirez-Manent J.I. (2025). Associations between shift work, sociodemographic and lifestyle characteristics, body measurements, and MASLD. Life.

[40] Xing X., Ding M., Li C. (2024). Combined effects of sleep timing and nighttime sleep duration on non-alcoholic fatty liver disease. Prev Med.

[41] Córdoba J., Cabrera J., Lataif L., Penev P., Zee P., Blei A.T. (1998). High prevalence of sleep disturbance in cirrhosis. Hepatology.

[42] Montagnese S., Middleton B., Mani A.R., Skene D.J., Morgan M.Y. (2010). On the origin and the consequences of circadian abnormalities in patients with cirrhosis. Am J Gastroenterol.

[43] Asher G., Gatfield D., Stratmann M. (2008). SIRT1 regulates circadian clock gene expression through PER2 deacetylation. Cell.

[44] Oike H., Oishi K., Kobori M. (2014). Nutrients, clock genes, and chrononutrition. Curr Nutr Rep.

[45] Stokkan K.-A., Yamazaki S., Tei H., Sakaki Y., Menaker M. (2001). Entrainment of the circadian clock in the liver by feeding. Science.

