# Supplementary material for: Circadian and behavioral timing in MASLD: a systematic review of human observational studies
Source: eClinicalMedicine. 2026 Aug 29;99:104187. doi: 10.1016/j.eclinm.2026.104187 (PMC13545344; doi:10.1016/j.eclinm.2026.104187)
Supplement: Supplementary file 1 [file mmc1.pdf]

Circadian and Behavioral Timing in MASLD: A Systematic Review of Human Observational Studies

Supplementary Material

Table of content

Supplementary Table 1: Eligibility criteria used for study selection
Supplementary Table 2: Search strategy – PubMed
Supplementary Table 3: Search strategy – Scopus
Supplementary Table 4: Search strategy – Embase
Supplementary Table 5: AXIS item-level appraisal and review-level overall judgments for cross-sectional studies and analytic components
Supplementary Table 5A: Summary interpretation of recurrent AXIS findings across 18 cross-sectional studies and analytic components
Supplementary Table 6: Newcastle-Ottawa Scale (NOS) appraisal of cohort studies
Supplementary Table 7: Newcastle-Ottawa Scale (NOS) appraisal of case-control studies
Supplementary Table 8: Adapted GRADE certainty-of-evidence assessment by circadian and behavioural timing domain
Supplementary Table 9: Biological sex representation and availability of sex-stratified evidence across included studies
Supplementary Table 10: Contextual studies evaluating circadian abnormalities in established cirrhosis

Supplementary Table 1. Eligibility criteria used for study selection

Criterion	Included	Excluded
**Population**	Adult human participants from community, occupational, population-based, or clinical settings. General adult populations were eligible when a MASLD-, MAFLD-, NAFLD-, steatosis-, fibrosis-, cirrhosis-, or quantitative liver-fat outcome relevant to the review was assessed.	Paediatric populations and non-human populations. Adult cohorts restricted to viral, alcohol-associated, autoimmune, cholestatic, mixed, unspecified, or other non-MASLD liver disease were excluded from the primary synthesis unless MASLD-, MAFLD-, or NAFLD-specific results were separately extractable.
**Exposure framework**	Naturally occurring indicators of circadian or behavioural timing, including shift work or night work; chronotype; observed sleep timing and sleep regularity; social jetlag or other study-defined circadian-misalignment constructs; objective rest–activity or light-related rhythm measures; endogenous melatonin or related circadian biomarker patterns; and meal-timing disruption.	Studies focused only on non-timing sleep traits, such as general sleep duration, insomnia, sleep quality, or obstructive sleep apnea, unless a distinct timing, regularity, or circadian-misalignment exposure was separately analyzed. Non-standard labels were not treated as established chronobiological constructs unless their operational definition was sufficiently described.
**Comparator**	Lower or absent exposure, alternative exposure categories, or reference timing patterns as defined by the original study.	Studies without a clinically interpretable comparator structure for exposure–outcome interpretation, except descriptive-supportive studies retained for contextual value.
**Outcomes**	Prevalent or incident MASLD/MAFLD/NAFLD; hepatic steatosis; significant or advanced hepatic fibrosis; cirrhosis; other disease-severity outcomes; and quantitative liver-fat measures assessed using imaging, elastography, biopsy, validated administrative coding, or accepted disease-oriented indices. Advanced fibrosis or cirrhosis was eligible for the primary synthesis when attributed to MASLD/MAFLD/NAFLD or when relevant subgroup data were separately extractable.	Nonspecific liver-enzyme abnormalities without a disease-oriented fatty liver outcome. Mixed or non-MASLD cirrhosis without separately extractable metabolic steatotic liver-disease data was excluded from the primary exposure–outcome synthesis but could be used for contextual interpretation of reverse causality.

Criterion	Included	Excluded
**Study design**	Observational designs evaluating naturally occurring circadian or behavioural timing exposures, including cross-sectional, case-control, prospective or retrospective cohort, longitudinal, and nested observational analyses.	Randomized or non-randomized studies assigning circadian-targeted treatments, including time-restricted eating, intermittent fasting, melatonin, light-based interventions, sleep scheduling, or shift-schedule modification, were excluded from the primary synthesis because they estimate intervention efficacy rather than associations with naturally occurring exposure. Their potential relevance to modifiability, reversibility, and biological plausibility was acknowledged separately. Mendelian-randomization studies, bioinformatics studies, and transcriptomic or gene-expression studies without direct clinical exposure–outcome analysis were also excluded.
**Melatonin-specific handling**	Observational studies assessing endogenous melatonin were eligible when the biological specimen, clock time and conditions of sampling, light conditions where relevant, assay method and identification, analytical units, and physiological interpretation were sufficiently reported. Serial timed measurements, dim-light melatonin onset, or sufficiently dense 24-hour profiles were considered the most informative measures of circadian phase or rhythmicity.	Studies were excluded when sampling time or conditions were not reported, when the assay platform could not be identified or verified, or when an isolated absolute concentration could not be interpreted as a measure of circadian phase, amplitude, secretion, or rhythmicity. Absolute concentrations were not compared across different biological matrices, assay platforms, sampling times, or light conditions. Cross-sectional associations with fibrosis categories were not interpreted as evidence of fibrosis progression. Melatonin supplementation and other therapeutic interventions were excluded from the primary observational synthesis.
**Publication type**	Full-text original research articles.	Conference abstracts, narrative reviews, editorials, commentaries, letters without original data.

Criterion	Included	Excluded
**Clinical interpretability**	Studies had to provide sufficient information to determine the direction of association between exposure and liver outcome.	Studies based on composite constructs such as "circadian syndrome" that did not isolate a clinically interpretable circadian exposure.
**Overlapping reports**	Reports from overlapping populations were linked as one underlying evidence source. The most comprehensive report was used for population characteristics, while unique exposure or outcome analyses from companion reports were retained.	Counting overlapping reports or shared survey cycles as independent replication.

Full-text exclusion terminology: In the PRISMA flow diagram, "ineligible population or liver-disease etiology" refers to reports outside the eligible adult-human population framework and reports restricted to non-MASLD, mixed, or unspecified liver disease without separately extractable MASLD-related data. This category is distinct from "non-MASLD-related outcome," which refers to studies involving an otherwise eligible population that did not report a qualifying liver outcome. Each full-text report was assigned one primary exclusion reason to avoid double counting.

Chronobiological terminology: Chronotype refers to a relatively stable preference or tendency toward earlier or later timing of sleep and activity. Sleep timing refers to observed bedtime, wake time, or sleep midpoint; sleep regularity refers to day-to-day stability or variability in these measures; and social jetlag refers to differences between workday and free-day sleep timing. Circadian misalignment denotes discordance between endogenous circadian timing and behavioural or environmental schedules. The expression "irregular chronotype" was not used because sleep-schedule irregularity is conceptually distinct from chronotype.

Chronotype-shift-work distinction: Chronotype was treated as an individual, trait-like timing phenotype reflecting preferred earlier or later timing. Shift work was treated as an externally imposed occupational schedule that may alter sleep opportunity, light exposure, activity, food timing, stress, and social behaviour. Shift work was therefore not interpreted as a direct measure of endogenous circadian phase, and chronotype was not treated as an environmental exposure of equivalent intensity. Potential mismatch between chronotype and work schedule was considered clinically relevant but was not adequately tested in the included evidence.

Supplementary Table 2: Search strategy - PubMed

Framework	Search Terms	Search hits as of 14 July 2026
Population	("Metabolic Dysfunction-Associated Steatotic Liver Disease"[tiab] OR MASLD[tiab] OR "Metabolic Dysfunction-Associated Fatty Liver Disease"[tiab] OR MAFLD[tiab] OR "Non-alcoholic Fatty Liver Disease"[Mesh] OR "nonalcoholic fatty liver disease"[tiab] OR NAFLD[tiab] OR "fatty liver"[tiab] OR "non-alcoholic fatty liver disease"[tiab] OR "Metabolic dysfunction associated steatotic liver disease"[tiab] OR "Metabolic dysfunction associated fatty liver disease"[tiab] OR steatohepatitis[tiab] OR MASH[tiab] OR NASH[tiab] OR "nonalcoholic steatohepatitis"[tiab] OR "non-alcoholic steatohepatitis"[tiab] OR "Liver Cirrhosis"[Mesh] OR "liver fibrosis"[tiab] OR "advanced liver fibrosis"[tiab] OR "hepatic fibrosis"[tiab] OR "advanced hepatic fibrosis"[tiab] OR (("advanced fibrosis"[tiab] OR cirrho*[tiab]) AND (liver[tiab] OR hepatic[tiab])))	239,153
Exposure	("Circadian Rhythm"[Mesh] OR circadian OR "circadian rhythm" OR "circadian misalignment" OR "circadian disruption" OR chronotype OR "morningness-eveningness" OR "evening chronotype" OR "morning chronotype" OR eveningness OR morningness OR "social jetlag" OR "social jet lag" OR "shift work" OR shiftworker* OR "shift workers" OR melatonin* OR "night shift" OR "night-shift" OR "night work" OR "rotating shift" OR "rotating shifts" OR "rotating-shift" OR "sleep timing" OR "sleep regularity" OR "sleep irregularity" OR "sleep-wake" OR "sleep wake" OR "meal timing" OR "eating timing" OR "feeding time" OR "late eating" OR "night eating" OR "timing of food intake" OR "rest-activity rhythm*" OR actigraphy)	195,179
P AND E	(("Metabolic Dysfunction-Associated Steatotic Liver Disease"[tiab] OR MASLD[tiab] OR "Metabolic Dysfunction-Associated Fatty Liver Disease"[tiab] OR MAFLD[tiab] OR "Non-alcoholic Fatty Liver Disease"[Mesh] OR "nonalcoholic fatty liver disease"[tiab] OR NAFLD[tiab] OR "fatty liver"[tiab] OR "non-alcoholic fatty liver disease"[tiab] OR "Metabolic dysfunction associated steatotic liver disease"[tiab] OR "Metabolic dysfunction associated fatty liver disease"[tiab] OR steatohepatitis[tiab] OR MASH[tiab] OR NASH[tiab] OR "nonalcoholic steatohepatitis"[tiab] OR "non-alcoholic steatohepatitis"[tiab] OR "Liver Cirrhosis"[Mesh] OR "liver fibrosis"[tiab] OR "advanced liver fibrosis"[tiab] OR "hepatic fibrosis"[tiab] OR "advanced hepatic fibrosis"[tiab] OR (("advanced fibrosis"[tiab] OR cirrho*[tiab]) AND (liver[tiab] OR hepatic[tiab])))) AND (("Circadian Rhythm"[Mesh] OR circadian OR "circadian rhythm" OR "circadian misalignment" OR "circadian disruption" OR chronotype OR "morningness-eveningness" OR "evening chronotype" OR "morning chronotype" OR eveningness OR morningness OR "social jetlag" OR "social jet lag" OR "shift work" OR shiftworker* OR "shift workers" OR melatonin* OR "night shift" OR "night-shift" OR "night work" OR "rotating shift" OR "rotating shifts" OR "rotating-shift" OR "sleep timing" OR "sleep regularity" OR "sleep irregularity" OR "sleep-wake" OR "sleep wake" OR "meal timing" OR "eating timing" OR "feeding time" OR "late eating" OR "night eating" OR "timing of food intake" OR "rest-activity rhythm*" OR actigraphy))	979

Supplementary Table 3: Search strategy - Scopus

Framework	Search Terms	Search hits as of 14 July 2026
Population	TITLE-ABS-KEY("Metabolic Dysfunction-Associated Steatotic Liver Disease" OR MASLD OR "Metabolic Dysfunction-Associated Fatty Liver Disease" OR MAFLD OR "nonalcoholic fatty liver disease" OR NAFLD OR "fatty liver" OR "non-alcoholic fatty liver disease" OR "Metabolic dysfunction associated steatotic liver disease" OR "Metabolic dysfunction associated fatty liver disease" OR steatohepatitis OR MASH OR NASH OR "nonalcoholic steatohepatitis" OR "non-alcoholic steatohepatitis" OR "liver cirrhosis" OR "liver fibrosis" OR "advanced liver fibrosis" OR "hepatic fibrosis" OR "advanced hepatic fibrosis" OR (("advanced fibrosis" OR cirrho*) AND (liver OR hepatic)))	418,693
Exposure	(circadian OR "circadian rhythm" OR "circadian misalignment" OR "circadian disruption" OR chronotype OR "morningness-eveningness" OR "evening chronotype" OR "morning chronotype" OR eveningness OR morningness OR "social jetlag" OR "social jet lag" OR "shift work" OR shiftworker* OR "shift workers" OR "night shift" OR "night-shift" OR "night work" OR "rotating shift" OR "rotating shifts" OR "rotating-shift" OR melatonin OR "sleep timing" OR "sleep regularity" OR "sleep irregularity" OR "sleep-wake" OR "sleep wake" OR "meal timing" OR "eating timing" OR "feeding time" OR "late eating" OR "night eating" OR "timing of food intake" OR "rest-activity rhythm*" OR actigraphy)	778,814
P AND E	TITLE-ABS-KEY("Metabolic Dysfunction-Associated Steatotic Liver Disease" OR MASLD OR "Metabolic Dysfunction-Associated Fatty Liver Disease" OR MAFLD OR "nonalcoholic fatty liver disease" OR NAFLD OR "fatty liver" OR "non-alcoholic fatty liver disease" OR "Metabolic dysfunction associated steatotic liver disease" OR "Metabolic dysfunction associated fatty liver disease" OR steatohepatitis OR MASH OR NASH OR "nonalcoholic steatohepatitis" OR "non-alcoholic steatohepatitis" OR "liver cirrhosis" OR "liver fibrosis" OR "advanced liver fibrosis" OR "hepatic fibrosis" OR "advanced hepatic fibrosis" OR (("advanced fibrosis" OR cirrho*) AND (liver OR hepatic))) AND ALL(circadian OR "circadian rhythm" OR "circadian misalignment" OR "circadian disruption" OR chronotype OR "morningness-eveningness" OR "evening chronotype" OR "morning chronotype" OR eveningness OR morningness OR "social jetlag" OR "social jet lag" OR "shift work" OR shiftworker* OR "shift workers" OR "night shift" OR "night-shift" OR "night work" OR "rotating shift" OR "rotating shifts" OR "rotating-shift" OR melatonin OR "sleep timing" OR "sleep regularity" OR "sleep irregularity" OR "sleep-wake" OR "sleep wake" OR "meal timing" OR "eating timing" OR "feeding time" OR "late eating" OR "night eating" OR "timing of food intake" OR "rest-activity rhythm*" OR actigraphy)	7973

Supplementary Table 4: Search strategy - Embase

Framework	Search Terms	Search hits as of 14 July 2026
Population #1	'metabolic dysfunction-associated steatotic liver disease':ti,ab,kw OR masld:ti,ab,kw OR 'metabolic dysfunction-associated fatty liver disease':ti,ab,kw OR mafld:ti,ab,kw OR 'nonalcoholic fatty liver disease':ti,ab,kw OR nafld:ti,ab,kw OR 'fatty liver':ti,ab,kw OR 'non-alcoholic fatty liver disease':ti,ab,kw OR 'metabolic dysfunction associated steatotic liver disease':ti,ab,kw OR 'metabolic dysfunction associated fatty liver disease':ti,ab,kw OR steatohepatitis:ti,ab,kw OR mash:ti,ab,kw OR nash:ti,ab,kw OR 'nonalcoholic steatohepatitis':ti,ab,kw OR 'non-alcoholic steatohepatitis':ti,ab,kw OR 'liver cirrhosis':ti,ab,kw OR 'liver fibrosis':ti,ab,kw OR 'advanced liver fibrosis':ti,ab,kw OR 'hepatic fibrosis':ti,ab,kw OR 'advanced hepatic fibrosis':ti,ab,kw OR (('advanced fibrosis':ti,ab,kw OR cirrho*:ti,ab,kw) AND (liver:ti,ab,kw OR hepatic:ti,ab,kw)) OR 'nonalcoholic fatty liver disease'/exp OR 'steatohepatitis'/exp OR 'liver fibrosis'/exp OR 'liver cirrhosis'/exp	444,352
Exposure #2	'circadian rhythm'/exp OR circadian:ti,ab,kw OR 'circadian rhythm':ti,ab,kw OR 'circadian misalignment':ti,ab,kw OR 'circadian disruption':ti,ab,kw OR chronotype:ti,ab,kw OR 'morningness-eveningness':ti,ab,kw OR 'evening chronotype':ti,ab,kw OR 'morning chronotype':ti,ab,kw OR eveningness:ti,ab,kw OR morningness:ti,ab,kw OR 'social jetlag':ti,ab,kw OR 'social jet lag':ti,ab,kw OR 'shift work':ti,ab,kw OR shiftworker*:ti,ab,kw OR 'shift workers':ti,ab,kw OR melatonin*:ti,ab,kw OR 'night shift':ti,ab,kw OR 'night-shift':ti,ab,kw OR 'night work':ti,ab,kw OR 'rotating shift':ti,ab,kw OR 'rotating shifts':ti,ab,kw OR 'rotating-shift':ti,ab,kw OR 'sleep timing':ti,ab,kw OR 'sleep regularity':ti,ab,kw OR 'sleep irregularity':ti,ab,kw OR 'sleep-wake':ti,ab,kw OR 'sleep wake':ti,ab,kw OR 'meal timing':ti,ab,kw OR 'eating timing':ti,ab,kw OR 'feeding time':ti,ab,kw OR 'late eating':ti,ab,kw OR 'night eating':ti,ab,kw OR 'timing of food intake':ti,ab,kw OR 'rest-activity rhythm*':ti,ab,kw OR actigraphy:ti,ab,kw	213,402
P AND E	#1 AND #2	1,539

Supplementary Table 5. AXIS item-level appraisal and review-level overall judgments for cross-sectional studies and analytic components

Legend: + = criterion adequately met or low concern; ! = criterion partially met, insufficiently reported, or some concerns; − = criterion not met or high concern. AXIS items were interpreted qualitatively and were not summed to create a numerical score. The final column represents a review-author-derived overall judgment rather than a score generated by the AXIS instrument.

Author	Q1	Q2	Q3	Q4	Q5	Q6	Q7	Q8	Q9	Q10	Q11	Q12	Q13	Q14	Q15	Q16	Q17	Q18	Q19	Q20	Review-level overall judgment
Chatur et al., 2024	+	+	−	+	!	−	−	+	+	+	+	+	−	−	+	+	+	+	!	+	High
Che et al., 2024	+	+	−	+	!	!	−	+	+	+	+	+	!	−	+	+	+	+	+	+	Moderate
Chen et al., 2026	+	+	−	+	+	+	!	+	+	+	+	+	!	+	+	+	+	+	+	+	Moderate
Gu et al., 2023	+	+	−	+	+	+	!	+	!	+	+	+	!	+	+	+	+	+	+	+	Moderate
Hu et al., 2026	+	+	−	+	!	!	−	+	+	+	+	+	!	−	+	+	+	+	+	+	Moderate
Kani et al., 2023	+	+	−	+	−	−	−	+	+	+	+	+	−	−	+	+	+	+	+	+	High
Kim et al., 2022	+	+	−	+	!	!	−	+	+	+	+	+	!	−	+	+	+	+	+	+	Moderate
Maidstone et al., 2024	+	+	−	+	!	!	!	+	!	+	+	+	!	!	+	+	+	+	+	+	Moderate
Peng et al., 2025	+	+	−	+	!	!	−	+	+	+	+	+	!	−	+	+	+	+	+	+	Moderate
Vetrani et al., 2022	+	+	−	+	!	!	−	+	!	+	+	+	!	−	+	+	+	+	+	+	Moderate
Weng et al., 2021	+	+	−	+	+	+	!	+	+	+	+	+	!	+	+	+	+	+	+	+	Moderate
Wu et al., 2025 (cross-sectional component)	+	+	−	+	+	+	!	+	+	+	+	+	!	+	+	+	+	+	+	+	Moderate
Zhang et al., 2025	+	+	−	+	!	!	−	+	+	+	+	+	!	−	+	+	+	+	+	+	Moderate
Zhang et al., 2020	+	+	−	+	!	!	−	+	+	+	+	+	!	−	+	+	+	+	+	+	Moderate
Castelnuovo et al., 2023	+	+	−	+	!	!	−	+	+	+	+	+	!	−	+	+	+	+	+	+	Moderate
Tosoratto et al., 2025	+	+	−	+	!	!	−	+	!	+	+	+	!	−	+	+	+	+	+	+	Moderate
Xing et al., 2024	+	+	−	+	!	!	−	+	+	+	+	+	!	−	+	+	+	+	+	+	Moderate

Author	Q1	Q2	Q3	Q4	Q5	Q6	Q7	Q8	Q9	Q10	Q11	Q12	Q13	Q14	Q15	Q16	Q17	Q18	Q19	Q20	Review-level overall judgment
**Zong et al., 2025**	+	+	−	+	+	+	!	+	+	+	+	+	!	!	+	+	+	+	+	+	**Moderate**

AXIS question key.

1. Were the aims/objectives clear?
2. Was the study design appropriate?
3. Was the sample size justified?
4. Was the target/reference population clearly defined?
5. Was the sample frame appropriate?
6. Was the selection process likely to yield a representative sample?
7. Were measures undertaken to address non-responders?
8. Were risk factor and outcome variables measured appropriately?
9. Were the measurement tools valid/reliable or adequately described?
10. Was statistical significance/precision clearly reported?
11. Were the methods sufficiently described?
12. Were the basic data adequately described?
13. Was the response rate adequate or unlikely to introduce meaningful non-response bias?
14. Was information about non-responders described?
15. Were the results internally consistent?
16. Were results presented for all analyses described?
17. Were the discussion and conclusions justified by the results?
18. Were limitations of the study discussed?
19. Were funding sources/conflicts of interest declared?
20. Were ethics approval/consent procedures reported?

Interpretation notes: The AXIS instrument does not prescribe a total score or validated thresholds for low, moderate, or high risk of bias. Items were therefore not summed. Review-level judgments were based primarily on four critical areas: (1) sampling frame, selection, and representativeness; (2) non-response and availability of information about non-responders; (3) validity and reliability of exposure and outcome measurement; and (4) completeness, internal consistency, and appropriateness of reporting.

Low risk indicated no major concern in the critical areas and only minor reporting limitations. Moderate risk indicated concerns that reduced generalizability or interpretability but did not fundamentally undermine the results. High risk indicated major concerns across multiple critical areas or a fundamental limitation in comparative interpretation. Absence of a formal sample-size justification was common and was not sufficient on its own to produce a high-risk judgment.

AXIS does not include a dedicated domain for analytical control of confounding. Covariate selection and adjustment were therefore assessed separately during data extraction and narrative synthesis. Wu et al. contributed both prospective and cross-sectional analyses; its NHANES component was evaluated using AXIS, while its UK Biobank component was assessed separately using the Newcastle–Ottawa Scale. Weng et al. and Zong et al. were appraised separately because they used different exposure definitions and liver outcomes, but they were treated as one underlying NHANES evidence source in review-level counts.

Supplementary Table 5A. Summary interpretation of recurrent AXIS findings across 18 cross-sectional studies and analytic components

Methodological area	Relevant AXIS items	Item-level pattern	Interpretation for the synthesis
**Objectives, design, and target population**	Q1, Q2, Q4	All 18 studies adequately stated their aims, used an appropriate cross-sectional design, and clearly defined the target or reference population.	Basic study purpose and design reporting were generally strong.
**Sample-size justification**	Q3	No study provided a formal sample-size justification.	This was a common reporting limitation and was not treated as sufficient by itself to determine high risk.
**Sampling frame and representativeness**	Q5-Q7	Only 5/18 studies clearly met Q5 and Q6; no study clearly met Q7.	Limited population representativeness and inadequate handling of non-response were major sources of potential selection bias.
**Exposure and outcome measurement**	Q8-Q9	All 18 studies adequately described the principal exposure and outcome variables; 14/18 clearly supported measurement validity or reliability, while four had some concerns.	Measurement was generally acceptable, but self-reported timing exposures and indirect liver outcomes reduced interpretability in some studies.
**Statistical reporting and internal consistency**	Q10-Q12; Q15-Q18	All 18 studies adequately reported precision, methods, basic data, internal consistency, planned analyses, justified conclusions, and limitations.	The principal weaknesses were not basic statistical reporting but selection, representativeness, temporality, and confounding.
**Response rate and non-response information**	Q13-Q14	No study clearly met Q13; only 4/18 clearly described non-responders in Q14.	Non-response bias and incomplete reporting of participation were recurrent limitations.
**Funding, conflicts, and ethical reporting**	Q19-Q20	Funding or conflict-of-interest reporting was adequate in 17/18 studies, and ethical reporting was adequate in all 18.	Governance and ethical reporting were generally satisfactory.

Methodological area	Relevant AXIS items	Item-level pattern	Interpretation for the synthesis
**Overall review-level judgment**	—	Sixteen appraisals were judged moderate risk and two were judged high risk; no cross-sectional study was judged low risk.	Cross-sectional evidence was retained but interpreted with reduced weight relative to prospective evidence.

Note. Frequencies describe item-level appraisal patterns and are not numerical AXIS scores. They are provided to explain the recurrent strengths and limitations that informed the narrative synthesis.

Supplementary Table 6. Newcastle-Ottawa Scale (NOS) appraisal of cohort studies

Legend: ★ = criterion met; — = criterion not clearly met/not reported.

Study	S1	S2	S3	S4	C1	C2	O1	O2	O3	Total	Overall
**Fu et al., 2023**	★	★	★	★	★	★	★	★	—	**8/9**	**Low**
**Huang et al., 2023**	★	★	★	★	★	★	★	★	★	**9/9**	**Low**
**Lee and Lee, 2024**	★	★	★	★	★	★	★	★	—	**8/9**	**Low**
**Shi et al., 2025**	★	★	★	★	★	★	★	★	—	**8/9**	**Low**
**Wu et al., 2025 (prospective component)**	★	★	★	★	★	★	★	★	★	**9/9**	**Low**
**Xu et al., 2023**	★	★	★	★	★	★	★	★	★	**9/9**	**Low**
**Qin et al., 2024**	★	★	★	★	★	★	★	★	—	**8/9**	**Low**

NOS cohort item key.

- S1: Representativeness of exposed cohort
- S2: Selection of non-exposed cohort
- S3: Ascertainment of exposure
- S4: Outcome absent at start
- C1: Comparability: main confounders
- C2: Comparability: additional confounders
- O1: Outcome assessment
- O2: Follow-up long enough
- O3: Adequacy of follow-up

Note. Wu et al. (2025) was appraised separately by analytic component. The prospective UK Biobank component is presented here as a cohort study, while the cross-sectional NHANES analyses are presented in Supplementary Table 5. Qin et al. identified baseline NAFLD using the Fatty Liver Index and incident cirrhosis using linked administrative records; these limitations were considered in the narrative interpretation.

Supplementary Table 7. Newcastle-Ottawa Scale (NOS) appraisal of case-control studies

Legend: ★ = criterion met; — = criterion not clearly met/not reported.

Study	CC1	CC2	CC3	CC4	C1	C2	E1	E2	E3	Total	Overall
**Schaeffer et al., 2024**	★	★	★	★	★	—	★	★	—	**7/9**	**Moderate**

NOS case-control item key. CC1 = case definition adequate; CC2 = representativeness of cases; CC3 = selection of controls; CC4 = definition of controls; C1 = comparability: main confounders; C2 = comparability: additional confounders; E1 = ascertainment of exposure; E2 = same method for cases and controls; E3 = non-response rate.

Supplementary Table 8. Adapted GRADE certainty-of-evidence assessment by circadian and behavioural timing domain

Exposure domain	Main outcome judged	Starting certainty	Main reasons for downgrading/upgrading	Final certainty	Suggested interpretation
Shift work-related exposures	MASLD/NAFLD/MAFLD occurrence or incidence	Low	Observational evidence; heterogeneous shift-work definitions; variation from imaging-confirmed disease to surrogate risk indices; residual confounding; and sensitivity to adiposity adjustment. Shift work is an externally imposed, multidimensional occupational condition rather than a direct measure of endogenous circadian phase. Certainty was not downgraded further because this domain included multiple longitudinal studies, a generally consistent direction of association, and some exposure-response patterns.	Low	Shift work may be associated with higher MASLD-related risk. It represents an externally imposed, multidimensional occupational exposure rather than a direct measure of circadian phase; residual occupational, behavioural, socioeconomic, and metabolic confounding remains important.
Chronotype	MASLD/NAFLD/MAFLD occurrence, significant or advanced fibrosis, and incident cirrhosis	Low	Observational evidence; mostly self-reported chronotype; selected populations; one small fibrosis study with highly imprecise estimates; inconsistent findings for advanced outcomes; and a null chronotype-specific association with incident cirrhosis. Chronotype is a trait-like individual timing phenotype, and the included studies rarely evaluated mismatch between preferred timing and externally required schedules.	Low	Later chronotype may be associated with MASLD occurrence, but chronotype is an individual timing phenotype rather than an environmental circadian stressor. Evidence for advanced outcomes is inconsistent, and the importance of mismatch between chronotype and required schedules remains uncertain.

Exposure domain	Main outcome judged	Starting certainty	Main reasons for downgrading/upgrading	Final certainty	Suggested interpretation
Sleep timing, sleep regularity, and circadian-misalignment constructs beyond sleep duration	Prevalent NAFLD/MASLD/MAFLD and fibrosis	Low	Three independent cross-sectional evidence sources represented by four reports; self-reported timing exposures; overlapping NHANES cycles; heterogeneous definitions; residual confounding; and no prospective evidence.	Very low	Later sleep timing and greater sleep-schedule variability may be associated with prevalent MASLD and fibrosis, but the heterogeneous behavioural constructs, cross-sectional designs, and absence of prospective physiological phase assessment make the findings very uncertain.
Objective rest–activity, light, and circadian biomarker measures	Prevalent and incident MASLD and biopsy-characterized MASLD phenotypes	Low	More direct exposure measurement and some prospective evidence supported the domain; however, only three independent evidence sources were available, objective metrics and liver outcomes differed substantially, wrist light exposure was an imperfect proxy for ocular exposure, and interpretable melatonin-phase evidence was limited to one small case-control study.	Low	Objective rest–activity and light measures may be associated with MASLD-related outcomes, but MASLD-specific evidence concerning melatonin phase is limited to one small case-control study. The evidence does not establish an association between absolute melatonin concentration and longitudinal fibrosis progression.
Meal timing-related exposures	Incident or prevalent MASLD/NAFLD	Low	Sparse evidence; self-reported meal timing; limited adjustment for total energy intake, diet composition, and broader dietary patterns. Certainty was supported by one large prospective cohort but limited by the small number of contributing studies.	**Low**	Meal-timing disruption may be associated with MASLD, but more prospective evidence with detailed dietary adjustment is needed.

Note: GRADE was applied using an adapted exposure-domain approach because statistical pooling was not clinically appropriate. Certainty ratings reflect confidence in the direction, consistency, and interpretability of the reported observational associations within each domain; they do not indicate that an exposure causes MASLD or its progression and do not represent certainty in a pooled effect estimate. The evidence matrix and harvest plot were descriptive and did not constitute meta-analysis. As all included studies were observational, each domain started at low certainty. Downgrading considered risk of bias, inconsistency, indirectness, imprecision, and suspected publication bias, whereas upgrading considered large magnitude of association, exposure-response patterns, and plausible residual confounding that would reduce the observed association. No domain was rated above low certainty because the evidence remained observational and was limited by residual confounding, heterogeneity, sparse study numbers, or cross-sectional designs. The domains represent distinct biological and behavioural constructs; certainty ratings should not be interpreted as comparative rankings of exposure intensity, causal importance, or clinical priority. In particular, shift work was treated as a multidimensional external schedule, whereas chronotype was treated as an individual timing phenotype.

Supplementary Table 9. Biological sex representation and availability of sex-stratified evidence across included studies

Study	Exposure domain	Population / design context	Sex composition	Handling of sex in the analysis	Sex-specific interpretation for this review
**Chatur et al., 2024**	Shift work	Rotating-shift security guards; cross-sectional occupational sample (India).	n=73; 47 male and 26 female.	No external daytime comparator; no sex-stratified exposure-outcome estimate was available.	Provides mixed-sex descriptive evidence from rotating shift workers, but it cannot support inference about sex differences in shift-work-related NAFLD risk.
**Che et al., 2024**	Shift work	Female nurses in a tertiary hospital; prediction-model analysis (China).	n=824; all participants were female nurses.	Female-only cohort; monthly night-shift frequency was included as a predictor; no male comparator.	Provides female occupational evidence, but findings should not be directly contrasted with male industrial cohorts because occupation, workload, shift pattern, and health behaviours may differ.
**Chen et al., 2026**	Sleep regularity / schedule discrepancy	US adults from NHANES; weekend catch-up sleep and prevalent MASLD.	Weighted mixed-sex sample; male 47.9% and female 52.1% in the overall weighted population.	Sex was included as an adjustment variable and examined in subgroup and interaction analyses; the original report labelled this variable as "gender." No statistically significant interaction by sex was reported.	Supports a sex-adjusted association between weekend catch-up sleep and MASLD, but does not provide strong evidence of sex-specific effect modification or direct evidence of endogenous circadian misalignment.
**Fu et al., 2023**	Chronotype	Employed adults in a longitudinal cohort from Southwestern China.	n=1,309; 1,259 male (96.2%) and 50 female (3.8%).	Models included sex; stratified analyses reported that age, sex, ethnicity, and education did not modify the chronotype-MAFLD association.	Useful because effect modification by sex was explicitly considered, although the cohort was highly male-predominant and therefore had limited female representation.
**Gu et al., 2023**	Objective rhythm / feeding rhythm	US adults with overweight/obesity from NHANES; 24-hour activity-rest and feeding-fasting rhythm indices.	n=4,502; female 51.26% overall; female 59.21% among suspected NAFLD cases.	Models adjusted for sex, which the original report labelled as "gender"; subgroup analyses focused mainly on obesity status rather than sex-specific estimates.	Provides mixed-sex evidence with covariate adjustment for sex, but it does not substantially clarify whether associations differ by sex.

Study	Exposure domain	Population / design context	Sex composition	Handling of sex in the analysis	Sex-specific interpretation for this review
**Hu et al., 2026**	Chronotype	Middle-aged and older adults; chronotype, metabolomics, and prevalent NAFLD (China).	n=744; 403 men (54.2%) and 341 women (45.8%). Men were more common in the NAFLD group.	Analyses adjusted for sex; effect modification by age, sex, and BMI was considered, but sex-specific results were not central to interpretation.	Shows reasonably balanced sex representation, but available evidence remains mainly sex-adjusted rather than sex-stratified for the chronotype-NAFLD association.
**Huang et al., 2023**	Shift work	UK Biobank prospective cohort of participants in paid or self-employment.	n=281,280; mixed-sex cohort. Night-shift workers were described as more likely to be male.	Models adjusted for sex; subgroup analyses stratified by sex were conducted, but sex-specific estimates were not emphasized in the main report.	Important large-cohort evidence for shift work and incident NAFLD, but sex-specific interpretation remains limited without detailed reporting of sex-stratified estimates.
**Kani et al., 2023**	Chronotype / sleep quality / night eating	Biopsy-proven MASLD clinical cohort (Turkiye).	n=93; 63 male (67.7%) and 30 female (32.3%).	Sex was described in baseline characteristics. PSQI score was higher in female than male participants; no significant sex difference was reported for the night-eating score.	Useful for identifying sex differences in sleep quality within a clinical MASLD cohort, but it does not establish modification of chronotype- or night-eating-related liver severity by sex.
**Kim et al., 2022**	Shift work	Steel manufacturing workers; cross-sectional study (South Korea).	n=2,511; all male workers. Women were excluded because no women were involved in shift work in the company.	Male-only analytic sample; no female comparator or sex-stratified estimate.	Provides male industrial shift-work evidence. Generalizability to female workers or mixed occupational settings is limited.
**Lee and Lee, 2024**	Shift work	Large longitudinal cohort of healthy Korean workers aged 20-59 years.	n=45,149; male 57.3% among daytime workers and 46.4% among shift workers.	Subgroup analyses were performed by sex. In the 20s age group, the shift-work association was statistically significant among women; no significant interaction was observed.	One of the more informative studies for sex-specific interpretation. Findings suggest a possible sex- and age-specific pattern, but the absence of significant interaction supports cautious interpretation.

Study	Exposure domain	Population / design context	Sex composition	Handling of sex in the analysis	Sex-specific interpretation for this review
**Maidstone et al., 2024**	Shift work / chronotype	UK Biobank employed adults; shift work, chronotype, DSI/ICD/MRI-PDFF liver phenotypes.	Current work schedule sample: day workers 46.01% male, irregular shift workers 53.39% male, permanent night-shift workers 61.1% male.	Models adjusted for sex; primary analyses were not presented as sex-stratified comparisons.	Shows sex imbalance across shift-work categories, which may contribute to residual heterogeneity. The study supports sex-adjusted associations but not sex-specific inference.
**Peng et al., 2025**	Shift work	Subway workers in Wuhan; cross-sectional occupational study (China).	n=9,105; 1,820 women (20.0%) and 7,285 men (80.0%). Participants with MAFLD were less often female (5.5%).	Models adjusted for sex; stratified analyses by sex were planned and interactions tested. Main-text heterogeneity was emphasized for job category and sleep duration, not sex.	The cohort was male-predominant and occupationally heterogeneous. Sex-specific inference is limited unless supplementary sex-specific estimates are extracted and interpreted.
**Schaeffer et al., 2024**	Objective rhythm / biomarker	Biopsy-proven MASLD clinical cohort and healthy controls; actigraphy and melatonin timing (Switzerland).	Healthy controls: 8 male/8 female; MASLD: 23 male/12 female. Subgroups varied in sex distribution.	No sex-stratified sleep or melatonin effect-modification analysis was reported in the main text.	Provides objective sleep/circadian phenotyping in a small mixed clinical sample, but the sample size limits sex-specific interpretation.
**Shi et al., 2025**	Meal timing	Kailuan prospective cohort; breakfast irregularity and late-night snacking (China).	n=32,030; 25,750 male (80.39%) and 6,280 female (19.61%).	Models adjusted for sex; subgroup analyses included sex and reported no significant interaction by sex for the main meal-timing exposures.	Large prospective evidence for meal timing, but the cohort was male-predominant. The reported lack of sex interaction should be interpreted cautiously in light of imbalance.
**Vetrani et al., 2022**	Chronotype	Adults with obesity and indirect NAFLD/NASH indices (Italy).	n=87; 64 female (73.6%) and 23 male (26.4%).	Analyses adjusted for sex, which the original study labelled as “gender,” together with age and BMI; no sex-stratified results were reported.	Female-predominant clinical obesity cohort. Findings support a sex-adjusted chronotype association but do not establish whether associations differ by sex.

Study	Exposure domain	Population / design context	Sex composition	Handling of sex in the analysis	Sex-specific interpretation for this review
**Weng et al., 2021**	Sleep timing / sleep regularity / study-defined misalignment	NHANES 2017-2018 population-based cross-sectional analysis with propensity-score matching.	Eligible sample n=4,552; 49.32% male. Before matching, CM participants were more likely male (55.82% vs 47.73%). Matched sample: 894 CM and 892 non-CM.	Propensity-score matching included age and sex; the original study labelled the latter variable as "gender." No sex-stratified association was reported.	Controls for sex through matching but does not clarify whether the study-defined sleep timing and regularity composite–MAFLD association differs between male and female participants.
**Wu et al., 2025**	Objective rhythm / light exposure	US NHANES and UK Biobank cohorts with accelerometry-derived light and rest-activity metrics.	US NHANES: male 44.9% in no-MASLD and 51.3% in prevalent MASLD. UK Biobank: male 43.4% in no-MASLD and 47.2% in incident MASLD.	Models adjusted for sex; subgroup analyses were planned by sex and other variables, with multiplicative interactions tested.	Strong objective-exposure study with mixed-sex representation, but sex-specific findings were not emphasized in the main report; detailed subgroup data would be needed for sex-specific interpretation.
**Xu et al., 2023**	Shift work	Chinese rail workers followed for 4 years.	n=14,112; 11,477 male (81.3%) and 2,635 female (18.7%). NAFLD incidence was higher in males than females in baseline characteristics.	Models adjusted for sex; stratified analyses suggested that shift-work-related NAFLD risk estimates appeared higher among female and older participants.	One of the most relevant studies for sex as a potential modifier, but female participants were a minority; findings should be treated as suggestive rather than definitive.
**Zhang J et al., 2025**	Shift work / occupational noise	Male automobile manufacturing workers; cross-sectional occupational study (China).	n=4,672; all male workers.	Male-only study; no female comparator; stratified analyses focused on age and occupational co-exposure.	Adds male industrial evidence but cannot inform sex differences. Occupational noise may also complicate attribution of shift-work effects.

Study	Exposure domain	Population / design context	Sex composition	Handling of sex in the analysis	Sex-specific interpretation for this review
**Zhang S et al., 2020**	Shift work	Steelworkers in North China; rotating night-shift exposure metrics.	n=6,881; predominantly male. Across NAFLD severity categories, the proportion male ranged from 89.4% to 97.4%.	Models adjusted for sex; sex-stratified analyses were reported. Male results were consistent with the overall analysis, whereas female estimates were directionally higher in some categories but not statistically significant.	Provides useful sex-stratified context, but the small number of female workers limited precision. Female estimates should be interpreted cautiously rather than as evidence of absence or presence of effect.
**Castelnuovo et al., 2023**	Chronotype	Adults with NAFLD in a cross-sectional Italian clinical cohort.	n=126; 73 male and 53 female.	Sex was included in multivariable models; no sex-stratified chronotype–fibrosis estimates were reported.	The mixed-sex clinical sample improves representation, but the study was too small to determine whether the chronotype–fibrosis association differed by sex.
**Qin et al., 2024**	Chronotype	UK Biobank participants with baseline NAFLD followed prospectively for incident cirrhosis.	Large mixed-sex cohort; the proportion female ranged from 39.3% to 47.5% across healthy-sleep-score categories.	Models adjusted for sex and menopausal status; subgroup analyses included sex.	The large cohort allowed sex-adjusted and subgroup analyses, but no robust sex-specific chronotype association with incident cirrhosis was established.
**Tosoratto et al., 2025**	Shift work	Spanish occupational cross-sectional study using non-invasive MASLD and fibrosis-risk indices.	Mixed-sex occupational cohort (n=53,053); the primary report included analyses by sex.	Sex was included in multivariable models and results were presented separately for male and female participants.	Provides sex-specific occupational context, but surrogate outcomes and the cross-sectional design limit conclusions regarding differential vulnerability by sex.
**Xing et al., 2024**	Sleep timing	Large cross-sectional study of adults attending medical-examination centres in China.	Mixed-sex sample of 39,471 adults.	Models adjusted for sex; interaction analyses suggested a stronger association of delayed sleep timing with NAFLD among male participants.	Suggests possible effect modification by sex, but the cross-sectional finding requires prospective confirmation.

Study	Exposure domain	Population / design context	Sex composition	Handling of sex in the analysis	Sex-specific interpretation for this review
**Zong et al., 2025**	Sleep timing / social jetlag / sleep debt	NHANES 2017–March 2020 cross-sectional analysis of sleep timing, MASLD, and liver fibrosis.	Mixed nationally representative sample (n=5,368).	Models adjusted for sex and included stratified analyses; some sleep-duration associations appeared more evident among female participants.	Adds sex-stratified context to the overlapping NHANES evidence, but timing-specific effect modification by sex was not established conclusively.

Abbreviations: CM, study-defined circadian-misalignment composite; DSI, Dallas Steatosis Index; MAFLD, metabolic dysfunction-associated fatty liver disease; MASLD, metabolic dysfunction-associated steatotic liver disease; NAFLD, nonalcoholic fatty liver disease; NHANES, National Health and Nutrition Examination Survey; NR, not reported or not clearly emphasized in the main report; PSQI, Pittsburgh Sleep Quality Index.

Terminology notes: This review uses "sex" for male and female participant categories and reserves "gender" for gender identity, gender roles, gender relations, or other socially constructed factors. Some original reports labelled a binary male/female variable as "gender"; because these studies did not measure gender identity or related social constructs, the variable is described as sex in this review. No included study explicitly assessed gender identity, gender roles, or gender relations.

Supplementary Table 10. Contextual studies evaluating circadian abnormalities in established cirrhosis

Study	Population/design	Circadian assessment	Principal findings	Interpretive direction	Role in this review
**Córdoba et al., 1998**	Patients with established cirrhosis; sleep questionnaire assessment with actigraphy in a subset and clinical comparison groups.	Sleep quality, sleep–wake timing, actigraphy, and diurnal preference.	Cirrhosis was associated with frequent sleep disturbance, delayed bedtime and wake time, and a greater tendency toward evening timing.	Established cirrhosis was associated with altered sleep–wake timing and organization.	Contextual evidence for reverse causality; excluded from primary MASLD exposure-domain counts and certainty ratings because the cirrhosis population was not restricted to MASLD.
**Montagnese et al., 2010**	Twenty patients with cirrhosis and nine healthy controls in a controlled circadian-physiology study.	Hourly plasma melatonin and cortisol over 24 hours, urinary 6-sulfatoxymelatonin, nocturnal light-suppression testing, actigraphy, and sleep diaries.	Patients showed delayed melatonin and cortisol peaks and reduced light-induced melatonin suppression; mean 24-hour melatonin clearance did not differ significantly between groups.	Established cirrhosis was associated with delayed circadian phase and reduced melatonin responsiveness to nocturnal light.	Contextual evidence for reverse causality and melatonin physiology; excluded from primary MASLD counts because the cirrhosis population was mixed-aetiology.

***Note**. These studies were used only to contextualize directionality, circadian physiology, and reverse causality and were not counted among the 24 independent MASLD-related evidence sources. Their findings concern sleep–wake timing, circadian phase, hormonal profiles, and responsiveness to light and should not be interpreted as demonstrating that an isolated absolute melatonin concentration predicts fibrosis severity or progression. Differences in biological matrix, sampling time, light conditions, assay method, and liver-disease severity preclude direct comparison of absolute melatonin values across studies.*
